# Nutrient Additions Regulate Height Growth Rate but Not Biomass Growth Rate of Alpine Plants Through the Contrasting Effect of Total and Available Nitrogen

**DOI:** 10.3390/plants14071143

**Published:** 2025-04-06

**Authors:** Runfang Feng, Shu Wang, Jikui Ma, Nannan Wang, Xiaoli Wang, Fei Ren, Honglin Li, Defei Liang, Jing Hu, Xilai Li, Lanping Li

**Affiliations:** 1 State Key Laboratory of Plateau Ecology and Agriculture, Qinghai University, Xining 810016, China; fengrf1018@163.com (R.F.); wangs9908@163.com (S.W.); majikui2022@163.com (J.M.); m18219969251@163.com (N.W.); wxl.yu@163.com (X.W.); fei_ren2016@163.com (F.R.); honglinli@qhu.edu.cn (H.L.); liangdefei-go@163.com (D.L.); xilai-li@163.com (X.L.); 2 Sanjiangyuan Grassland Ecosystem National Observation and Research Station, Xining 810016, China; 3 College of Agriculture and Animal Husbandry, Qinghai University, Xining 810016, China; 4 College of Agriculture and Forestry Sciences, Qinghai University, Xining 810016, China; 5 College of Landscape Architecture and Life Science, Institute of Special Plants, Chongqing University of Arts and Sciences, Chongqing 402160, China; 9986hujing@163.com

**Keywords:** nitrogen and phosphorus addition, relative growth rate, biomass, seasonal dynamic, Qinghai–Tibet plateau, alpine plant

## Abstract

Plant growth, a fundamental biological process that underpins terrestrial ecosystem function, is susceptible to nutrient availability. Despite extensive research on lowland ecosystems, the responses of alpine plant growth to nutrient addition remain poorly understood, particularly given the heightened sensitivity of alpine ecosystems to global change. To investigate the effects of nitrogen (N) and phosphorus (P) additions on the growth rates of alpine plants and the underlying mechanisms of how these nutrient additions influence plant growth rates, we conducted an experiment in an alpine grassland on the Qinghai–Tibet Plateau, targeting 14 common plant species. Growth rates were measured using biomass and height, with plant height and soil physicochemical properties recorded biweekly during the growing season. We assessed the effects of nitrogen and phosphorus additions on growth rates, their seasonal dynamics, and their relationships with soil physicochemical properties. Results showed that phosphorus addition and combined nitrogen-phosphorus additions significantly increased the relative growth rate based on height (RGR_H_). In contrast, nutrient additions had no significant effect on the relative growth rate based on biomass (RGR_B_). RGR_H_ decreased from June and early July to August, exhibiting species-specific responses to nutrient additions. Additionally, RGR_H_ was significantly influenced by the interaction of nitrogen and phosphorus additions, species, and seasonal dynamics (*p* < 0.05). Soil available N, available P, and moisture were significantly positively correlated with RGR_H_ (*p* < 0.05), while soil temperature (ST), total nitrogen (TN), and soil organic carbon (SOC) exhibited significant negative correlations (*p* < 0.05). Nutrient additions altered the hierarchy, as well as the direct and indirect factors that influence RGR_H_, revealing the opposing regulatory effects of total and available nitrogen. These findings highlight the critical roles of nitrogen and phosphorus, suggesting phosphorus is a potential limiting factor for plant growth in this alpine region. This study offers a comprehensive analysis of how nitrogen and phosphorus additions affect alpine plant growth rates and clarifies the underlying mechanisms in these sensitive ecosystems.

## 1. Introduction

Plant growth is a fundamental biological process that underpins terrestrial ecosystem function. It is defined as the combination of irreversible and reversible changes in biomass and volume, including cell production, structural development, and expansion [1]. Early studies laid the groundwork for our understanding of plant growth. De Saussure [2] demonstrated that growth depends on water uptake and carbon fixation, while Sprengel [3] and Liebig [4] highlighted the importance of soil nutrient uptake. Plant growth is essential for the success of any plant species and is closely tied to ecological strategies and crop productivity, which form the basis for food supply and USD 4.3 trillion of agricultural production in 2020 of agricultural production [5,6]. For instance, Hancock [7] explored the relationship between cotton plant growth and yield, and ecological theories such as r/K selection [8] and CSR strategies [5] developed from principles of plant growth have furthered our understanding of growth strategies across environmental gradients. At the individual level, growth is vital for survival and reproduction, as it optimizes resource acquisition, such as light interception and root exploration [9]. At the community level, plant growth influences interspecific interactions, community assembly, and ecological succession by enabling transitions from pioneer to secondary communities through differential resource use and tolerance [10]. As the cornerstone of ecosystems, plant growth supports critical functions like carbon sequestration, energy flow, and nutrient cycling, thereby bolstering ecosystem resilience [11].

Variations in plant growth arise from both genetic background and environmental conditions such as light intensity, temperature, soil moisture, and nutrient availability [12]. For example, in alpine ecosystems, low temperatures and short growing seasons restrict plant growth and reproduction [13]. Alpine plants often exhibit specialized physiological and morphological adaptations, such as compact growth patterns and enhanced cold tolerance, compared to those found at lower altitudes [5]. In terrestrial ecosystems, nitrogen and phosphorus are often the primary limiting factors for plant growth [14,15,16]. Studies indicate that nitrogen frequently limits plant growth at high latitudes, while phosphorus constrains growth at low latitudes [17], although some studies suggest that a combination of nitrogen and phosphorus can co-limit growth [16]. At present, atmospheric nitrogen and phosphorus deposition are increasing rapidly due to global climate change and intensified human activities, which can partially alleviate nutrient limitations [18,19,20]. However, excessive deposition may lead to nutrient imbalances in the soil. Previous research on plant growth primarily focuses on growth dynamics in crops and horticultural species. However, the effects of environmental factors, particularly nitrogen and phosphorus availability, on alpine plant growth remain understudied. Alpine plants play a crucial role in the ecological balance of alpine ecosystem through water conservation, carbon sequestration, soil conservation, and maintaining biodiversity [21]. At the same time, support for plateau animal husbandry, the development of medicinal resources, and eco-tourism value based on alpine plants is becoming a strategic resource for the sustainable development of the alpine region [22]. Moreover, research on alpine plants has mainly examined changes in biomass rather than growth rates. For example, Fu and Shen [23] and Shen et al. [24] reported that nitrogen addition increases biomass in alpine grasslands. Similarly, Wang et al. [25] found significant increases in the community biomass of Poaceae, Cyperaceae, and Fabaceae following phosphorus fertilization. Despite the potentially profound effects of increasing global atmospheric nitrogen and phosphorus deposition on plant growth, little is known about how nutrient additions affect the growth rates of alpine plants [26].

The relative growth rate (RGR) is widely employed in quantifying plant growth dynamics. RGR quantifies the net increase in biomass or dimensions over time and represents the proportional increase relative to the initial size, thereby eliminating biases from ontogenetic stage differences and enabling comparisons across species or environments [27]. Unlike biomass measurements, height assessments do not require plant destruction, enabling repeated measurements on the same individual to track growth over time [9]. As a key determinant of light competition and resource acquisition, particularly in dense vegetation, height is a practical and meaningful indicator for studying plant growth dynamics [28]. This methodological framework is especially important for alpine ecosystems—environments defined by brief growing seasons and harsh climatic conditions that amplify the sensitivity of growth dynamics crucial for forecasting plant responses to environmental disturbances, such as variations in nutrient availability and the effects of climate change.

Given these methodological advantages and the urgent need to understand how alpine plants respond to global change, quantifying the role of nutrient-mediated growth modulation remains surprisingly overlooked—an issue that becomes particularly significant when we consider the unique biogeochemical drivers of high-altitude ecosystems. To address this knowledge gap regarding the ways nutrient additions affect the growth rates of alpine plants, we conducted a nutrient addition experiment in an alpine grassland on the Qinghai–Tibet Plateau. We selected fourteen common plant species and measured their growth rates using biomass and height metrics. Additionally, we recorded plant height and soil physicochemical properties biweekly during the growing season to examine growth rate dynamics and uncover the mechanisms by which nutrient additions influence alpine plant growth rates. Specifically, we investigated the following questions: (1) How do nitrogen and phosphorus additions affect alpine plant growth rates? (2) What are the species-specific and seasonal variations in growth rates under nutrient additions, and do they interact? (3) What are the underlying mechanisms, including the direct and indirect effects of soil physicochemical factors, through which nutrient additions influence growth rates? We hypothesize that the response of alpine plant growth rates depends on the nutrient limitations in the study area. We expect species-specific and seasonal variations in growth rates due to nutrient additions, although their interactions remain uncertain. Furthermore, we anticipate that nutrient additions influence growth rates through both the direct and indirect effects of soil physicochemical factors.

## 2. Results

### 2.1. Response of the Growth Rate of Alpine Plants to Nutrient Additions

**Figure 1:**
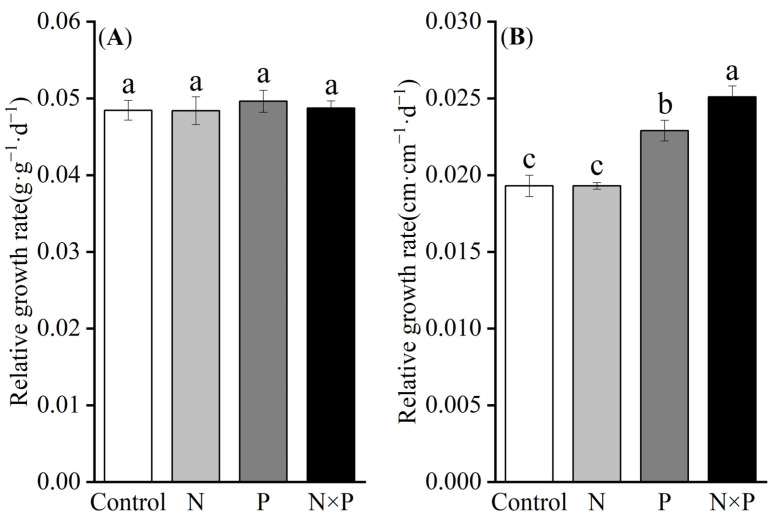
Effect of nutrient additions on the relative growth rate of alpine plants. Control represents the control; N denotes nitrogen addition; P signifies phosphorus addition; N × P indicates the combined addition of nitrogen and phosphorus; (**A**) shows the effect of nitrogen and phosphorus additions on RGR_B_; (**B**) illustrates the effect of nitrogen and phosphorus additions on RGR_H_; different lower-case letters indicate significant differences between the various nutrient addition treatments (*p* < 0.05).

One-way ANOVA results indicated that nutrient addition did not significantly affect the relative growth rate based on the biomass (RGR_B_) of alpine plants (*p* > 0.05, Figure 1A). However, phosphorus addition alone and the combined addition of nitrogen and phosphorus significantly increased the relative growth rate based on height (RGR_H_) (*p* < 0.05), while nitrogen addition alone had no significant effect on RGR_H_ (Figure 1B). Since nutrient additions did not significantly influence RGR_B_, it was excluded from further analysis.

### 2.2. Response of the Growth Rate of Different Species to Nutrient Additions

The RGR_H_ of different species responded differently to nutrient additions. For five species—*Poa versicolor*, *Thermopsis lanceolata*, *Elymus nutans*, *Medicago ruthenica*, and *Saussurea nigrescens*—phosphorus addition alone and the combined addition of nitrogen and phosphorus significantly increased their RGR_H_ (*p* < 0.05, Table 1). The RGR_H_ of *Ranunculus membranaceus* was significantly increased only by the phosphorus addition alone (*p* < 0.05, Table 1). The RGR_H_ of *Aster diplostephioides*, *Galium boreale*, and *Saussurea pulchra* was significantly increased only by the combined addition of nitrogen and phosphorus (*p* < 0.05, Table 1). For *Anemone obtusiloba*, nitrogen addition alone significantly reduced the RGR_H_ (*p* < 0.05), while other nutrient additions had no significant effect (*p* > 0.05, Table 1). Nutrient additions did not significantly influence the RGR_H_ of *Morina chinensis*, *Potentilla saundersiana*, *Gentiana straminea*, or *Oxytropis kansuensis* (*p* > 0.05, Table 1).

### 2.3. Seasonal Dynamic of the Growth Rate of Alpine Plants Under Different Nutrient Additions

**Figure 2:**
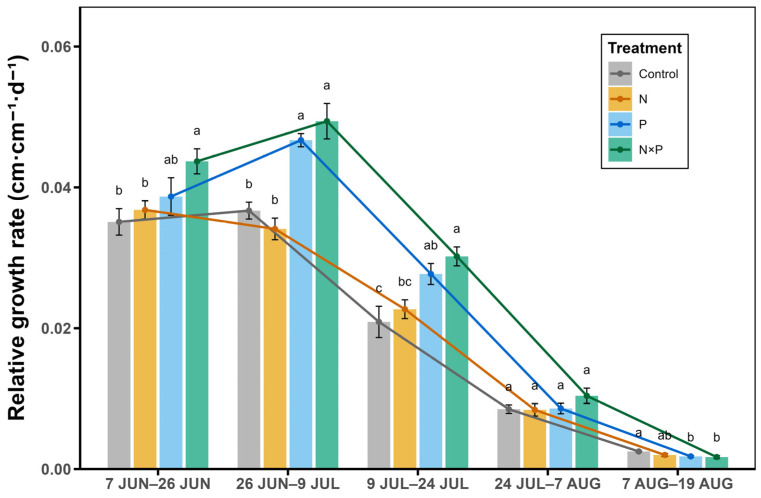
Effect of nitrogen and phosphorus added alone and in combination on the relative growth rate of plants at different growth stages. Lowercase letters on the error bars indicate significant differences under different treatments in that time period; 7 JUN–26 JUN indicates the relative growth rate from 7 June to 26 June; 26 JUN–9 JUL indicates the relative growth rate from 26 June to 9 July; 9 JUL–24 JUL indicates the relative growth rate from 9 July to 24 July; 24 JUL–7 AUG indicates the relative growth rate from 24 July to 7 August; and 7 AUG–19 AUG indicates the relative growth rate from 7 August to 19 August.

The RGR_H_ was relatively high in June and early July, and it then tended to decrease from that period into August (Figure 2). The combined addition of nitrogen and phosphorus significantly accelerated RGR_H_ in the first half of the growing season (*p* < 0.05, Figure 2), Phosphorus addition alone significantly accelerated the RGR_H_ from 26 June to 24 July (*p* < 0.05, Figure 2), while nitrogen addition alone had little influence on the RGR_H_ throughout the growing season (*p* > 0.05, Figure 2). The overall response of the RGR_H_ to nutrient additions during the growth and development process was in the following order: N × P > P > N.

Multifactorial ANOVA revealed a significant interaction effect of nitrogen addition, phosphorus addition, species, and seasonal dynamics on the RGR_H_ (Table 2). This suggests that the growth rates of alpine plants under nutrient addition varied by species and time. Therefore, when analyzing the effect of nutrient additions on alpine plant growth rates, the influence of species and month should be considered.

### 2.4. Relationship Between Growth Rate and Soil Physical and Chemical Properties

**Figure 3:**
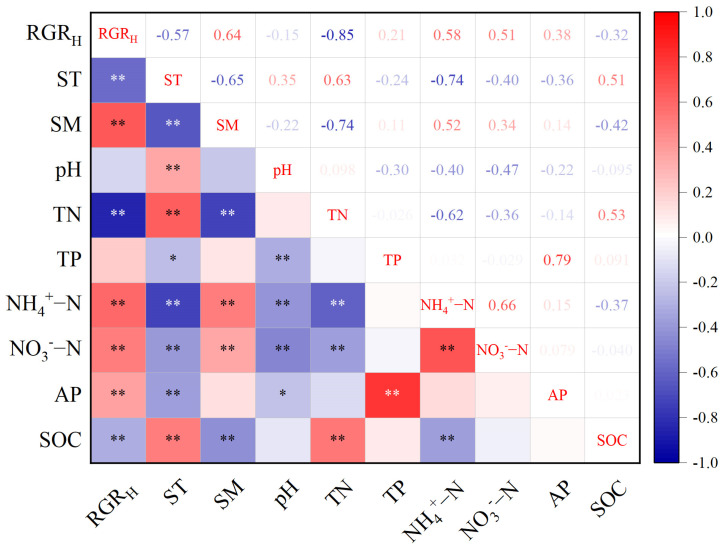
Pairwise correlation between soil physical and chemical factors and relative growth rate. Correlations are indicated by different colors (*, *p* < 0.05; **, *p* < 0.01), with positive correlations in red, negative correlations in blue, and color gradients representing the strength of the correlation. RGR_H_ denotes relative growth rate; ST denotes soil temperature; SM denotes soil moisture; pH denotes soil pH; TN denotes total nitrogen content; TP denotes total phosphorus content; NH_4_^+^-N indicates ammonium nitrogen content; NO_3_^−^-N indicates nitrate nitrogen content; AP denotes available phosphorus content; and SOC denotes soil organic carbon content.

Correlation analysis showed that soil moisture (SM), ammonium nitrogen (NH_4_^+^-N), nitrate nitrogen (NO_3_^−^-N), and available phosphorus (AP) were significantly positively correlated with the RGR_H_ (*p* < 0.05), while soil temperature (ST), total nitrogen (TN), and soil organic carbon (SOC) were significantly negatively correlated with the RGR_H_ (*p* < 0.05). Soil pH and total phosphorus (TP) showed no significant correlation with the RGR_H_ (Figure 3).

### 2.5. Direct and Indirect Effects of Soil Physical and Chemical Properties on Plant Growth

**Figure 4:**
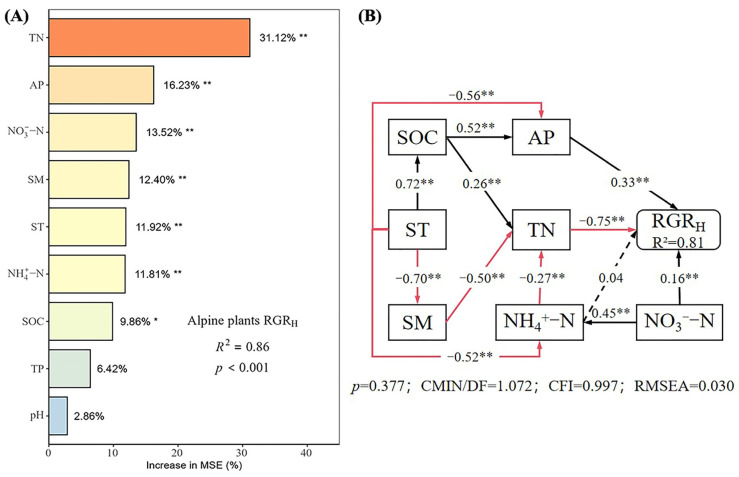
Importance and effect of soil physicochemical factors on the relative growth rate. (**A**) represents the result of the random forest model; (**B**) represents the result of the structural equation model. (**A**) Different color depths in the plot indicate the relative importance of soil factors to RGRH, with darker colors indicating higher importance and lighter colors indicating lower importance; (**B**) The black solid line in the figure indicates a significant positive correlation; the red solid line indicates a significant negative correlation; the black dashed line indicates a non-significant positive correlation; the red dashed line indicates a non-significant negative correlation; * indicates *p* < 0.05; ** indicates *p* < 0.01.

The random forest model explained 86% of the variation in the RGR_H_ (Figure 4A), identifying TN, AP, NO_3_^−^-N, SM, ST, NH_4_^+^-N, and SOC as the main factors affecting the RGR_H_. The SEM explained 81% of the variance in the RGR_H_ (Figure 4B). AP, NO_3_^−^-N, and NH_4_^+^-N had direct positive effects on the RGR_H_, with path coefficients of 0.33, 0.16, and 0.04, respectively, though the effect of NH_4_^+^-N was not significant. TN had a significant direct negative effect on the RGR_H_ (*p* < 0.05), with a path coefficient of −0.75. Additionally, SOC, ST, SM, NO_3_^−^-N, and NH_4_^+^-N had indirect effects on the RGR_H_. Overall, the total effects on RGR_H_ were ranked as SM (0.38) > AP (0.33) > NH_4_^+^-N (0.24) > NO_3_^−^-N (0.18) > SOC (0.02) > ST (−0.41) > TN (−0.75).

**Figure 5:**
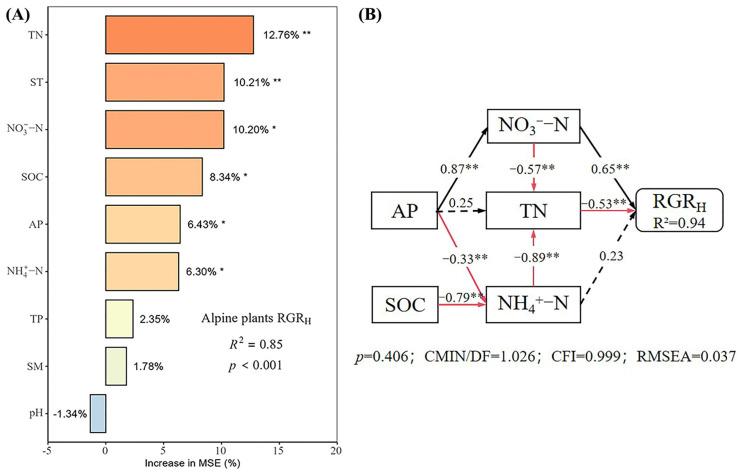
Importance and effect of soil physicochemical factors on the RGR_H_ with control. (**A**) represents the result of the random forest model; (**B**) represents the result of the structural equation model. * indicates *p* < 0.05; ** indicates *p* < 0.01.

**Figure 6:**
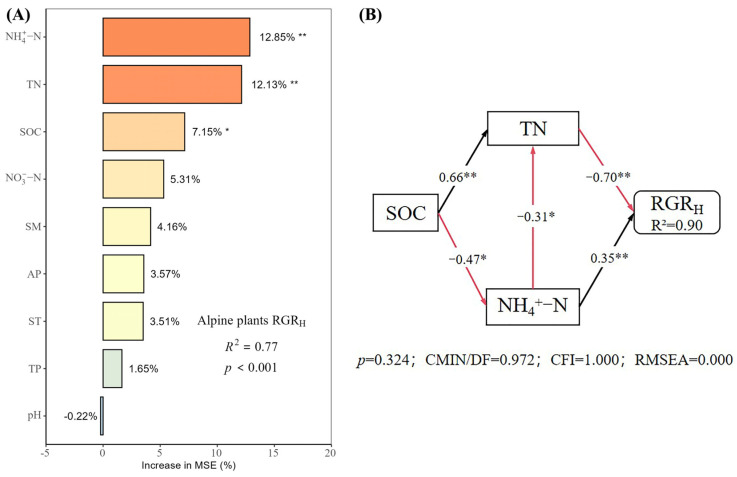
Importance and effect of soil physicochemical factors on the RGR_H_ with nitrogen treatment. (**A**) represents the result of the random forest model; (**B**) represents the result of the structural equation model. * indicates *p* < 0.05; ** indicates *p* < 0.01.

**Figure 7:**
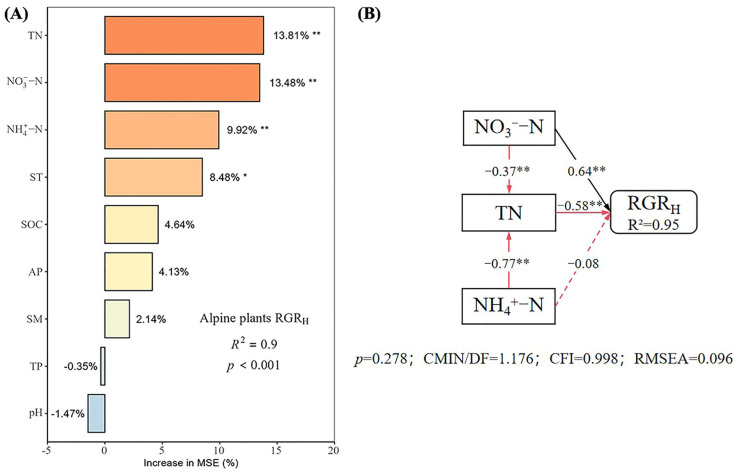
Importance and effect of soil physicochemical factors on the RGR_H_ with phosphorus treatment. (**A**) represents the result of the random forest model; (**B**) represents the result of the structural equation model. * indicates *p* < 0.05; ** indicates *p* < 0.01.

**Figure 8:**
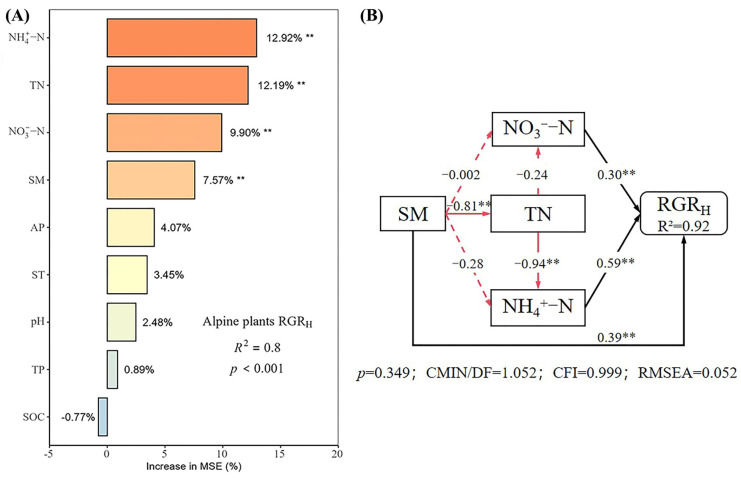
Importance and effect of soil physicochemical factors on the RGR_H_ with nitrogen and phosphorus treatment. (**A**) represents the result of the random forest model; (**B**) represents the result of the structural equation model. ** indicates *p* < 0.01.

Under different nutrient additions, random forest models explained 85%, 77%, 90%, and 80% of the variation in the RGR_H_ for the control, N, P, and N × P treatments, respectively (Figure 5, Figure 6, Figure 7 and Figure 8). In the control treatment (Figure 5A), TN, ST, NO_3_^−^-N, SOC, AP, and NH_4_^+^-N were the main factors influencing the RGR_H_. For N addition (Figure 6A), NH_4_^+^-N, TN, and SOC were the primary factors. In P addition (Figure 7A), TN, NO_3_^−^-N, NH_4_^+^-N, and ST were the main factors, while in N × P addition (Figure 8A), NH_4_^+^-N, TN, NO_3_^−^-N, and SM had more potent effects.

The results of the structural equation modeling under different nutrient additions showed that in the control treatment (Figure 5B), the model explained 94% of the variation in the RGR. Among them, NO_3_^−^-N and TN produced significant direct effects on the RGR_H_ (*p* < 0.05), but TN produced negative effects, with impact path coefficients of 0.65 and −0.53, respectively. NH_4_^+^-N also had a direct effect on the RGR_H_, but the effect was not significant. In addition, AP and SOC had indirect effects on the RGR_H_. In the N-alone addition treatment (Figure 6B), the model explained 90% of the variation in the RGR. Among them, NH_4_^+^-N and TN had a significant direct effect on the RGR_H_ (*p* < 0.05) and a negative effect on TN with impact path coefficients of 0.35 and −0.70, respectively. In addition, SOC has an indirect effect on the RGR through NH_4_^+^-N and TN. In the P-alone addition treatment (Figure 7B), the model explained 95% of the variation in the RGR_H_. The direct significant factors were the same as the control, and NO_3_^−^-N produced a significant direct positive effect (*p* < 0.05). TN produced a significant direct negative effect (*p* < 0.05), with impact path coefficients of 0.64 and −0.58, respectively, whereas NH_4_^+^-N produced a non-significant direct negative and indirect effect on the RGR_H_. In the N × P addition treatment (Figure 8B), the model explained 92% of the variation in the RGR. Among them, NO_3_^−^-N, NH_4_^+^-N, and SM exerted significant direct positive effects (*p* < 0.05) on the RGR_H_, with influence path coefficients of 0.30, 0.59, and 0.39, respectively, whereas TN and SM also affected the RGR_H_ indirectly via NO_3_^−^-N and NH_4_^+^-N.

Overall, TN negatively affected the RGR_H_ under both nutrient addition and control conditions, except in the N × P treatment, where it did not directly affect the RGR_H_. Additionally, the RGR_H_ was primarily influenced by NH_4_^+^-N under N treatment, by NO_3_^−^-N under P treatment and control, and by both NH_4_^+^-N and NO_3_^−^-N under N × P treatment. Among all factors, SOC influenced RGR_H_ only under N treatment and control, AP influenced RGR_H_ only under control, and SM influenced RGR_H_ only with the N × P treatment.

## 3. Discussion

Alpine plant growth and development are significantly constrained by soil nutrient availability, particularly nitrogen and phosphorus [13]. The atmospheric deposition of these nutrients can enrich the soil but may also cause imbalances, influencing plant growth [26]. We examined how plant responses to nutrient additions provide insights into the future growth trajectories of alpine species. Our results indicate that nutrient additions affected the height growth rate more than the biomass growth rate, with alpine plant height being particularly sensitive to phosphorus addition. We also observed species-specific and seasonal variations and their interaction with growth rates under nutrient additions, which highlighted the complexity of these responses. Additionally, nutrient additions altered the hierarchy of direct and indirect factors affecting plant growth, showcasing the opposing regulatory effects of total and available nitrogen. This study elucidates how nitrogen and phosphorus additions impact alpine plant growth, offering insights into the future growth trajectories of alpine plants amidst global change.

The addition of nutrients had contrasting effects on the biomass-based relative growth rate (RGR_B_) and height-based relative growth rate (RGR_H_). While nutrient additions did not significantly alter the RGR_B_, they significantly increased RGR_H_. This result aligns with our previous work, which found that nutrient additions did not significantly affect biomass but did enhance the height of alpine plants [29]. This suggests that the increase in the community’s biomass did not stem from changes in individual biomass but may instead arise from density or species composition [30]. The discrepancy may originate from a decrease in stem tissue mass density or from resource allocation patterns, whereby alpine plants prioritize flowering over vegetative biomass accumulation, considering that the sampling period coincided with the reproductive phase [29]. Phosphorus addition alone, as well as the combined addition of nitrogen and phosphorus, significantly increased the relative growth rate based on height (RGR_H_), with the latter yielding the most pronounced effect, consistent with prior research [31,32]. However, this contrasts with findings that nitrogen addition enhances aboveground and individual biomass in goatgrass communities, which may indicate differences in nutrient limitation or experiment conditions between the study areas [33]. This result suggests that height growth in alpine plants is particularly sensitive to phosphorus addition, underscoring the role of phosphorus in facilitating vertical expansion and alleviating nutrient constraints in the studied species [34,35]. We can predict a higher yet consistent biomass in the plant communities of the study area if more phosphorus or both nitrogen and phosphorus are introduced in the future.

Our results revealed species-specific variations in RGR_H_ responses to nutrient additions. Nitrogen addition influenced the RGR_H_ of only one species, while phosphorus addition enhanced the RGR_H_ of six species. Furthermore, the combined addition of nitrogen and phosphorus accelerated the RGR_H_ of eight species. Among these, the RGR_H_ of Poaceae and Fabaceae was mainly affected by phosphorus addition and the combined nitrogen-phosphorus addition. The combined nitrogen-phosphorus addition had a greater effect on the RGR_H_ of Forbs than the other nutrient additions. Thus, most of the selected species may be primarily impacted by phosphorus limitation or nitrogen-phosphorus co-limitation, which aligns with some research findings [34,35], though it contradicts reports of nitrogen-driven height increases [36]. These variations may reflect discrepancies in the environmental factors of the study area or species identification. The species-specific variations in RGR_H_ responses to nutrient additions may correlate strongly with their light competition ability, ultimately influencing their population dynamics. Notably, Poaceae exhibited the most significant height increases, potentially contributing to their dominance in fertilized plots [37].

Seasonal dynamics further modulated the RGR_H_, with peak growth observed in the early growing season, followed by a decline consistent with the natural growth cycles of alpine species [38]. The response hierarchy (N × P > P > N) also indicates phosphorus limitation or nitrogen-phosphorus co-limitation in the study area. Specifically, the effects of phosphorus addition alone and the combined nitrogen and phosphorus addition on the RGR_H_ diminished in the late growing season. This outcome highlights the significance of the time on growth rates, particularly in ecosystems with short growing seasons. Furthermore, the results of the multifactorial ANOVA confirmed significant interactions among nitrogen, phosphorus, species identity, and seasonal timing (*p* < 0.05), underscoring the complexity of these responses. It is essential to consider comprehensive conditions when studying plant growth, especially in the field. Here, we might obtain entirely different results if we select fewer species or assess them in the later growing season.

Plant–soil interactions are crucial in alpine ecosystems. Correlation analyses revealed that soil moisture (SM), ammonium nitrogen (NH_4_^+^-N), nitrate nitrogen (NO_3_^−^-N), and available phosphorus (AP) were significantly positively correlated with the RGR_H_ (*p* < 0.05). In contrast, soil temperature (ST), total nitrogen (TN), and soil organic carbon (SOC) showed significant negative correlations with the RGR_H_ (*p* < 0.05). These findings suggest that in these alpine ecosystems, readily available nutrients contribute to plant growth more effectively than the total nutrient pools. This conclusion reinforces earlier observations by Vitousek et al. [39] that nutrient bioavailability, rather than merely the presence of nutrients, is critical for plant performance. As noted in previous studies, these available nutrients promote plant growth by enhancing nutrient uptake efficiency and supporting photosynthetic capacity [40]. Specifically, NH_4_^+^-N and NO_3_^−^-N facilitate nitrogen assimilation, while AP meets the phosphorus demands essential for vertical growth [34]. Soil moisture plays a particularly influential role in regulating nutrient availability in the moisture-limited alpine environment [13], with random forest modeling identifying it as the strongest predictor of RGR_H_ (total effect = 0.38). These patterns align with Luo et al. [41], who found that soil moisture and available nutrients are essential for plant growth in moisture-limited alpine regions. Moreover, this finding is consistent with the results of Winkler et al., who suggest that the effect of temperature on alpine productivity is largely dependent on soil moisture content during the growing season. Increased temperature reduces soil moisture, which in turn lessens the positive effects on plant growth [42]. In contrast, the present study found that soil temperature decreases with nutrient additions, leading to an increase in soil moisture. This decrease in temperature is accompanied by an accumulation of Poaceae biomass, which contains a significant amount of polymers, including lignin, that may help maintain cellular water potential and buffer fluctuations in microenvironmental humidity [43,44]. The negative impact of TN suggests that excessive total nitrogen accumulation may lead to nutrient imbalances or unfavorable soil conditions—such as acidification—that inhibit plant growth despite the presence of mineral nitrogen forms [41]. Similarly, SOC’s negative correlation may indicate its indirect effect on nutrient availability through microbial activity, particularly under heightened TN conditions [45]. The inverse relationship between ST and RGR_H_ could be attributed to temperature-induced phenological delays or its antagonistic interaction with SM [46]. These findings emphasize that readily available nutrients, rather than total nutrient pools, primarily drive alpine plant growth, aligning with observations from Vitousek et al. [39].

Random forest modeling identified TN, AP, NO_3_^−^-N, SM, ST, NH_4_^+^-N, and SOC as key predictors of the RGR_H_, with SM exerting the most substantial total effect (0.38). This finding reflects the intricate interplay of natural conditions and highlights the critical role of soil moisture in mediating nutrient availability and plant growth in alpine ecosystems, where water stress is prevalent [13]. The dominance of SM in our models suggests that climate-driven changes in precipitation could significantly influence nutrient cycling and plant performance, amplifying the ecological significance of our results. In the absence of nutrient additions (control treatment), TN, ST, NO_3_^−^-N, SOC, AP, and NH_4_^+^-N emerge as the primary factors influencing the RGR_H_. Under nitrogen addition, NH_4_^+^-N, TN, and SOC gain prominence, indicating that nitrogen inputs enhance the role of available nitrogen [23]. With phosphorus addition, TN, NO_3_^−^-N, NH_4_^+^-N, and ST dominate, suggesting that phosphorus alters nitrogen uptake dynamics. In the combined N × P treatment, NH_4_^+^-N, TN, NO_3_^−^-N, and SM become the leading factors, with the increased significance of SM underscoring its mediation of nutrient cycling under synergistic conditions [47]. This variability across treatments illustrates how nutrient additions reshape the drivers of plant growth by modifying nutrient availability and plant uptake preferences.

Notably, the dominant form of nitrogen shifts with treatment as follows: NO_3_^−^-N prevails in control and phosphorus addition scenarios, while NH_4_^+^-N takes precedence under nitrogen addition, with both forms significantly contributing under N × P treatment. This flexibility in nitrogen utilization highlights the adaptive responses to nutrient enrichment [48]. Additionally, nitrogen addition may induce phosphorus limitation, whereas N × P treatments mitigate the negative effects of TN, enhancing growth through phosphorus availability [49]. Although phosphorus does not have a direct effect on plant growth, a deficiency in phosphorus can lead to reductions in both root and shoot development. For example, under low phosphorus conditions, soybean leaves may turn yellow; these yellow leaves can gradually change to red and then purple, ultimately resulting in the wilting of the plant [50]. In addition, it has been shown that phosphorus deficiency also delays the initiation of plant rhizome function and reduces rhizome development, leading to impaired symbiotic nitrogen fixation. Therefore, phosphorus deficiency, along with other elements, limits the growth of plants [51]. In addition to changes in intrinsic plant factors affecting growth, extrinsic factors such as climate warming may further regulate phosphorus bioavailability. Environmental factors may also affect intrinsic plant responses, triggering signals for resource acquisition when the plant perceives unfavorable conditions. This perception tends to decrease the rate of resource acquisition; for example, adverse ground conditions often trigger changes in the balance between abscisic acid, cytokinin, and gibberellins, thereby impacting plant growth. Consequently, unfavorable environmental conditions generally lead to reduced plant growth rates [12]. SEM further clarifies the mechanisms underlying these relationships. A striking discovery was the opposing regulatory effects of TN and available nitrogen on the RGR_H_. SEM analysis indicated a strong negative direct effect of TN on RGR_H_, supporting the notion that high total nitrogen may induce nutrient imbalances or promote conditions unfavorable for growth. In contrast, AP, NO_3_^−^-N, and NH_4_^+^-N exerted positive direct effects on the RGR_H_, with a more substantial effect from NH_4_^+^-N attributed to its higher uptake efficiency [52]. In the N × P treatment, this negative TN effect diminishes, and growth is primarily driven by the available nitrogen, with phosphorus enhancing nitrogen utilization efficiency [49]. This interplay between TN and available nitrogen forms highlights a nuanced regulatory mechanism where the benefits of nutrient availability and balanced uptake offset the detrimental effects of total nutrient accumulation. SOC influences the RGR_H_ indirectly through its effects on nitrogen and phosphorus availability, a role that is more pronounced in the control and nitrogen addition treatments due to microbial sensitivity [45]. Soil moisture’s significant contribution to the N × P treatment reinforces its regulatory role in nutrient dynamics [53]. These findings suggest that soil conditions mediate nutrient availability and uptake, shaping plant growth responses in alpine grasslands.

The integrated findings of this study have significant ecological implications for nutrient-poor alpine ecosystems. Firstly, the evident positive influence of available nutrients and soil moisture on relative growth rates highlights the essential role of resource availability in driving plant performance. The importance of soil moisture indicates that climate-driven changes in precipitation could considerably affect plant growth in the alpine ecosystem. Secondly, the contrasting effects of total nitrogen and available nitrogen imply that excessive nitrogen accumulation can disrupt the nutrient balance, possibly through microbial interactions, resulting in reduced growth even when mineral forms of nitrogen are present. Lastly, the ability of phosphorus to relieve these nutrient constraints—both directly and indirectly—emphasizes its central role in supporting plant productivity and facilitating vertical expansion in nutrient-limited alpine systems. These findings offer a foundation for managing alpine plant growth in the context of global change, advocating for strategies that incorporate moisture availability, nutrient interactions, and balanced nutrient inputs to improve ecosystem stability and function.

This study examined how nitrogen and phosphorus additions affect the physicochemical properties of soil and their impact on the growth rates of alpine plants. The results revealed that the effects of various nutrient additions on alpine plant growth rates differ, as do the impacts of different soil physicochemical characteristics under nutrient addition. However, plant growth is primarily limited by nitrogen, where total soil nitrogen negatively influences plant growth, while ammonium and nitrate nitrogen have a positive effect. Additionally, available soil phosphorus, organic carbon, temperature, and humidity also play significant roles in plant growth. In conclusion, in light of the global atmospheric nitrogen and phosphorus deposition, this study provides foundational data for predicting future changes in alpine grassland development and for mitigating the adverse effects of nitrogen enrichment on the growth and development of alpine plants. However, the growth of alpine plants is a complex process. Based on the results of this study, future analyses should integrate changes in the climate, shifts in phytochemical characteristics, multi-gradient and multi-nutrient additions, and other multidimensional analyses to explore the comprehensive mechanisms of alpine plant growth under varying environmental conditions.

## 4. Materials and Methods

### 4.1. Study Area and Experimental Design

This study was conducted in an alpine grassland near the Haibei Alpine Grassland Ecosystem Research Station, Qinghai, China, on the Qinghai–Tibetan Plateau (37°39′ N, 101°19′ E, altitude 3231 m) (Figure S1). The climate is characterized by a continental monsoon with short, cool summers and long, cold winters. The average annual temperature is approximately −1.7 °C, with the coldest month (January) averaging −15.1 °C and the hottest month (July) averaging 9.8 °C. Annual precipitation ranges from 425.36 to 850.4 mm, with an average of 582.1 mm. Approximately 80% of the annual precipitation occurs during the growing season (May to September) [54]. The plant communities are dominated by *Kobresia humilis* and *Elymus nutans*, with common species including *Poa versicolor*, *Stipa aliena*, *Anemone obtusiloba*, *Thermopsis lanceolata*, *Medicago ruthenica*, *Gentiana straminea*, *Saussurea nigrescens*, *Saussurea pulchra*, and *Galium boreale*.

The experiment was designed as a randomized complete block design with four treatments as follows: control (no nutrient addition), nitrogen (N) addition (100 kg ha^−1^ year^−1^), phosphorus (P) addition (100 kg ha^−1^ year^−1^), and a combined N and P addition (100 kg ha^−1^ year^−1^ each). Four replications per treatment resulted in 16 plots (each 6 m × 6 m), with a 1 m buffer strip between each plot. Each plot was divided into two parts as follows: one for observing stationary individual plants and the other for collecting plant and soil samples. The experimental plots were fenced in 2017, and nutrient addition treatments began in 2018. Fertilizer was applied by evenly spreading pre-weighed granular fertilizer over the plots during the first and second weeks of June each year. Applications were timed before rain on cloudy days or in the evening on sunny days to facilitate rapid dissolution into the soil via rainfall or dew. Urea was used as the nitrogen source, and Triple Super Phosphate was used for phosphorus.

This study was conducted in 2023, the sixth year of fertilizer treatment, and focused on the 14 most common species in the plant community, as follows: *Elymus nutans*, *Poa versicolor*, *Thermopsis lanceolata*, *Oxytropis kansuensis*, *Anemone obtusiloba*, *Ranunculus membranaceus*, *Morina chinensis*, *Potentilla saundersiana*, *Medicago ruthenica*, *Saussurea pulchra*, *Saussurea nigrescens*, *Aster diplostephioides*, *Gentiana straminea*, and *Galium boreale*.

### 4.2. Sample Collection and Measurement

Five relatively intact, pest- and disease-free individuals of each selected species in each plot were labeled at the end of May. The height of each labeled individual was measured every two weeks until the end of August. Soil samples, consisting of three borings (0–10 cm depth) per plot, were collected from the soil sample collection area concurrently with plant height measurements. These samples were then brought back to the laboratory for soil property determination.

Aboveground parts of the selected species were collected in early June and at full bloom in each plot to measure biomass. Five intact individuals of each species, free of pests and diseases, were collected. The samples were dried at 65 °C for 48 h and then measured for biomass.

### 4.3. Determination of Soil Physical and Chemical Properties

WatchDog 1200 instruments (Spectrum, Aurora, IL, USA) were installed at the end of April to monitor soil temperature and moisture at 5 cm depth in the sample plots. Data were collected in October, the end of the growing season. Collected soil samples were sieved, air-dried, and used for various analyses. Soil pH was determined by adding distilled water and shaking. Ammonium and nitrate nitrogen were extracted using KCl solution, while available phosphorus was extracted with NaHCO_3_ solution. Additionally, soil samples were acid-digested to determine organic carbon, total nitrogen, and total phosphorus contents. Soil organic carbon was measured by titration, and soil ammonium nitrogen, nitrate nitrogen, available phosphorus, total nitrogen, and total phosphorus were analyzed using a SEAL AA3 AutoAnalyzer (SEAL Analytical, Wrexham, UK).

### 4.4. Calculation of the Plant Growth Rate

In this study, the relative growth rates (RGRs) were calculated based on both plant height (RGR_H_) and biomass (RGR_B_). The RGR_B_ was calculated using the aboveground biomass from the first sampling after fertilizer application and at full bloom. The RGR_H_ was calculated using the heights measured from June to August. The formula used was the following:

Relative Growth Rate (RGR) = (lnY_2_ − lnY_1_)/(t_2_ − t_1_)

where Y_1_ and Y_2_ are the biomass or height at times t_1_ and t_2_, respectively. Additionally, the RGR_H_ was calculated for each measurement period to examine the seasonal dynamics of the plant growth rate under different nutrient additions. The RGR is expressed in units per day. To facilitate calculations, all dates were converted to Julian days (the number of days since 1 January).

### 4.5. Data Analysis

All data were tested for normality and log-transformed if necessary. Data were analyzed using SPSS 26.0 and plotted using Origin 2021. One-way ANOVA, followed by the Least Significant Difference (LSD) test, was used to compare variations in plant growth rates among treatments using SPSS 26.0. A multivariate ANOVA was employed to investigate the interactive effects of nitrogen and phosphorus additions, species, and seasonal dynamics on plant growth rates using SPSS 26.0. Correlation analysis was conducted to examine the relationships between soil physical and chemical factors and plant growth rates using SPSS 26.0. Random forest analysis using R 4.3.2 was used to rank the importance of soil factors influencing plant growth rates. Soil factors significantly affecting plant growth rates were selected and checked for multicollinearity. Factors with high collinearity were excluded, and the remaining factors were incorporated into structural equation models (SEM) to assess the effects of soil nutrients on plant growth rates under different nutrient addition scenarios using Amos 24.0.

## Figures and Tables

**Table 1:** Effect of nitrogen and phosphorus added alone and in combination on the RGR_H_ of 14 plant species.

Species	Control	N	P	N × P
*P. versicolor*	0.022 ± 0.001 c	0.025 ± 0.001 bc	0.027 ± 0.001 ab	0.031 ± 0.001 a
*A. obtusiloba*	0.028 ± 0.000 a	0.021 ± 0.002 b	0.027 ± 0.001 a	0.027 ± 0.001 a
*R. membranaceus*	0.017 ± 0.001 bc	0.016 ± 0.002 c	0.022 ± 0.002 a	0.022 ± 0.001 ab
*M. chinensis*	0.022 ± 0.003 a	0.023 ± 0.002 a	0.027 ± 0.001 a	0.025 ± 0.001 a
*T. lanceolata*	0.017 ± 0.001 b	0.016 ± 0.001 b	0.022 ± 0.000 a	0.024 ± 0.002 a
*E. nutans*	0.023 ± 0.002 b	0.022 ± 0.002 b	0.027 ± 0.001 a	0.031 ± 0.000 a
*P. saundersiana*	0.023 ± 0.001 a	0.021 ± 0.001 a	0.022 ± 0.002 a	0.026 ± 0.002 a
*M. ruthenica*	0.023 ± 0.001 b	0.024 ± 0.000 b	0.031 ± 0.001 a	0.032 ± 0.001 a
*S. nigrescens*	0.014 ± 0.005 b	0.018 ± 0.001 ab	0.025 ± 0.001 a	0.026 ± 0.002 a
*G. straminea*	0.019 ± 0.000 a	0.019 ± 0.001 a	0.016 ± 0.004 a	0.019 ± 0.001 a
*A. diplostephioides*	0.015 ± 0.001 b	0.018 ± 0.001 b	0.018 ± 0.002 b	0.024 ± 0.001 a
*G. boreale*	0.016 ± 0.001 b	0.016 ± 0.001 b	0.020 ± 0.002 b	0.026 ± 0.002 a
*S. pulchra*	0.013 ± 0.000 bc	0.011 ± 0.001 c	0.016 ± 0.001 ab	0.018 ± 0.002 a
*O. kansuensis*	0.021 ± 0.001 a	0.021 ± 0.001 a	0.023 ± 0.001 a	0.024 ± 0.001 a

Note: *P. versicolor* is Poa versicolor; *A. obtusiloba* is Anemone obtusiloba; *R. membranaceus* is Ranunculus membranaceus; *M. chinensis* is Morina chinensis; *T. lanceolata* is Thermopsis lanceolata; *E. nutans* is Elymus nutans; *P. saundersiana* is Potentilla saundersiana; *M. ruthenica* is Medicago ruthenica; *S. nigrescens* is Saussurea nigrescens; *G. straminea* is Gentiana straminea; *A. diplostephioides* is Aster diplostephioides; *G. boreale* is Galium boreale; *S. pulchra* is Saussurea pulchra; and *O. kansuensis* is Oxytropis kansuensis; different lower-case letters indicate significant differences between the various nutrient addition treatments (*p* < 0.05).

**Table 2:** Effect of nitrogen and phosphorus additions, species, and time on RGR_H_.

Factors	df	F	*p*
N addition	1	6.586	0.010 *
P addition	1	117.313	0.000 **
N addition × P addition	1	5.939	0.015 *
N addition × P addition × Species	52	6.443	0.000 **
N addition × P addition × Time	16	282.104	0.000 **
N addition × P addition × Time × Species	208	3.822	0.000 **

Note: * indicates *p* < 0.05; ** indicates *p* < 0.01.

## Data Availability

Data are included in the Supplementary Materials (Tables S1 and S2).

## Supplementary Materials

The following supporting information can be downloaded at: https://www.mdpi.com/article/10.3390/plants14071143/s1, Figure S1: Location map of the test area; Figures S2–S10: Seasonal dynamics of soil physicochemical factors; Table S1: Alpine plant growth rate data; Table S2: Seasonal dynamics of soil physicochemical properties.
