# Supplementary material for: Nutrient Additions Regulate Height Growth Rate but Not Biomass Growth Rate of Alpine Plants Through the Contrasting Effect of Total and Available Nitrogen
Source: Plants (Basel). 2025 Apr 6;14(7):1143. doi: 10.3390/plants14071143 (PMC11991464; doi:10.3390/plants14071143)
Supplement: Supplementary file 1 [file plants-14-01143-s001.zip › Figure S1-S10.pdf]

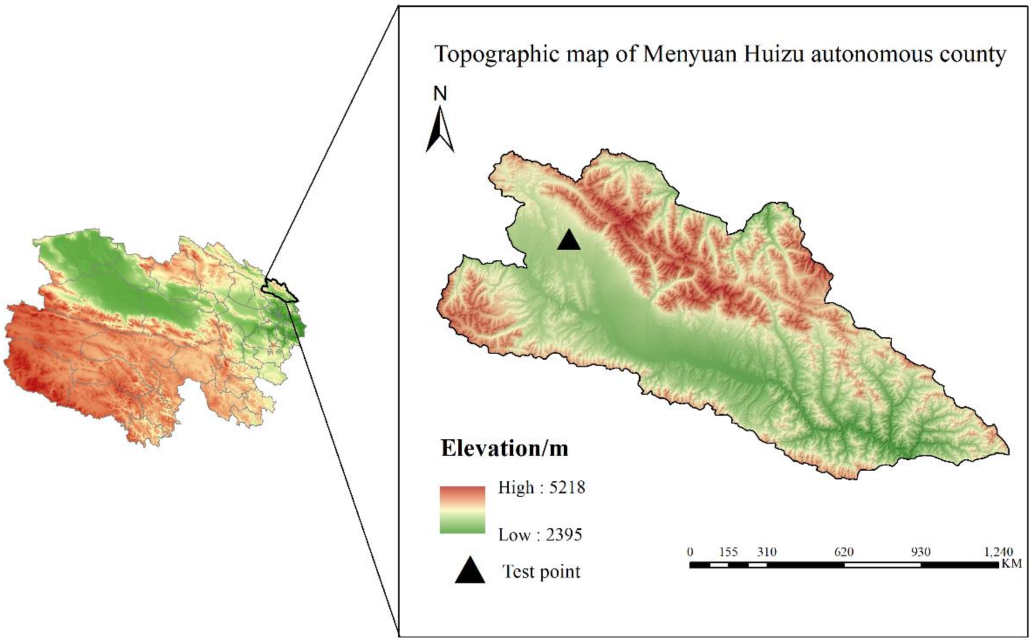

Figure S1. Location map of the study area

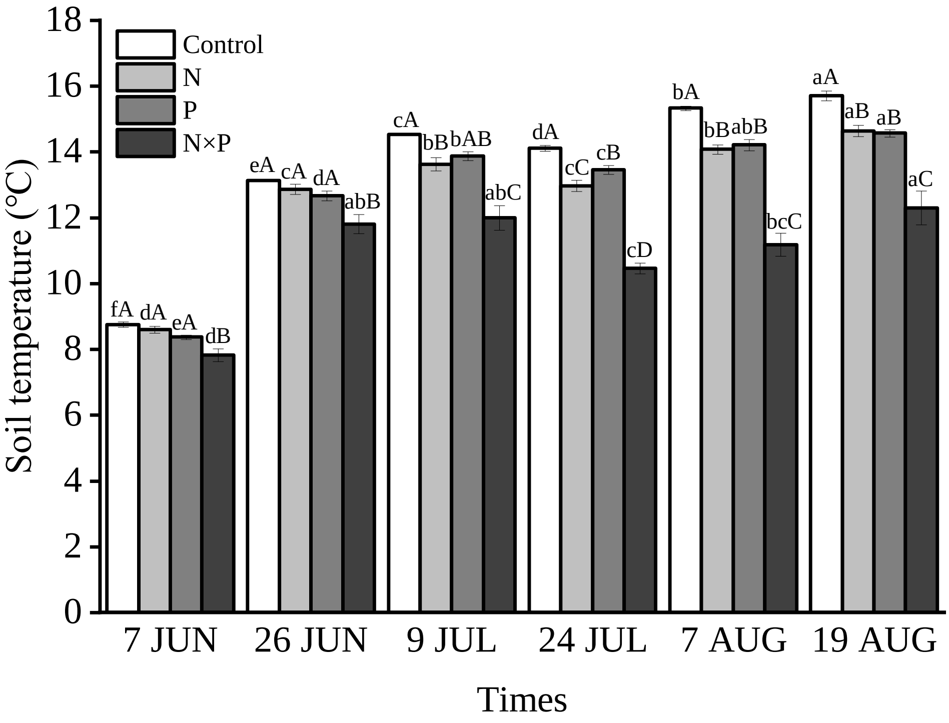

Figure S2 Seasonal changes in soil temperature with different nutrient additions

Note: Control indicates control; N indicates nitrogen-added treatment; P indicates phosphorus-added treatment; and N×P indicates mixed nitrogen-phosphorus-added treatment; 7 JUN indicates soil sampling data from June 7th; 26 JUN indicates soil sampling data from June 26th; 9 JUL indicates soil sampling data from July 9th; 24 JUL indicates soil sampling data from July 24th; 7 AUG indicates soil sampling data from August 7th; 19 AUG indicates soil

sampling data from August 19th; Different lowercase letters indicate significant differences in soil properties at different times; different uppercase letters indicate significant differences between treatments at the same time (P<05); the same below.

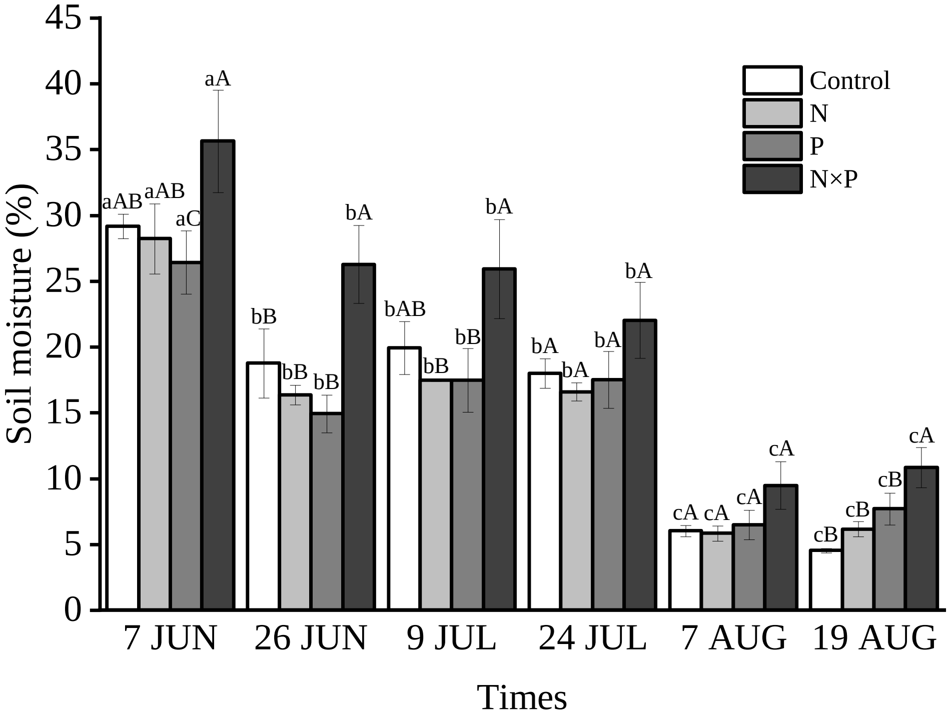

Figure S3 Seasonal variation of soil moisture under different nutrient additions

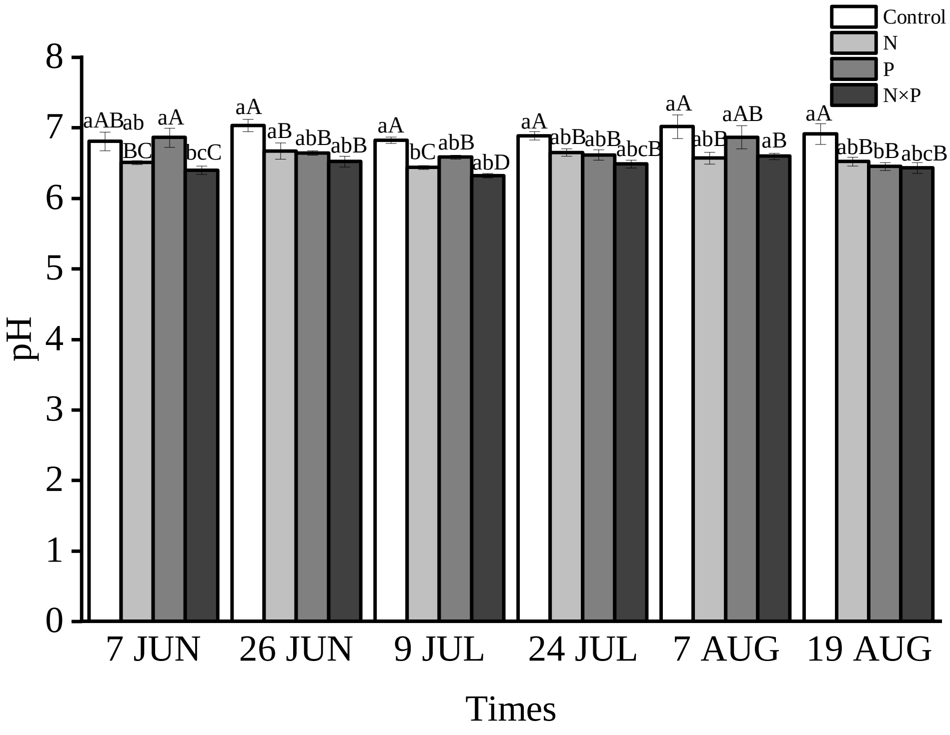

Figure S4 Seasonal variation of soil pH under different nutrient additions

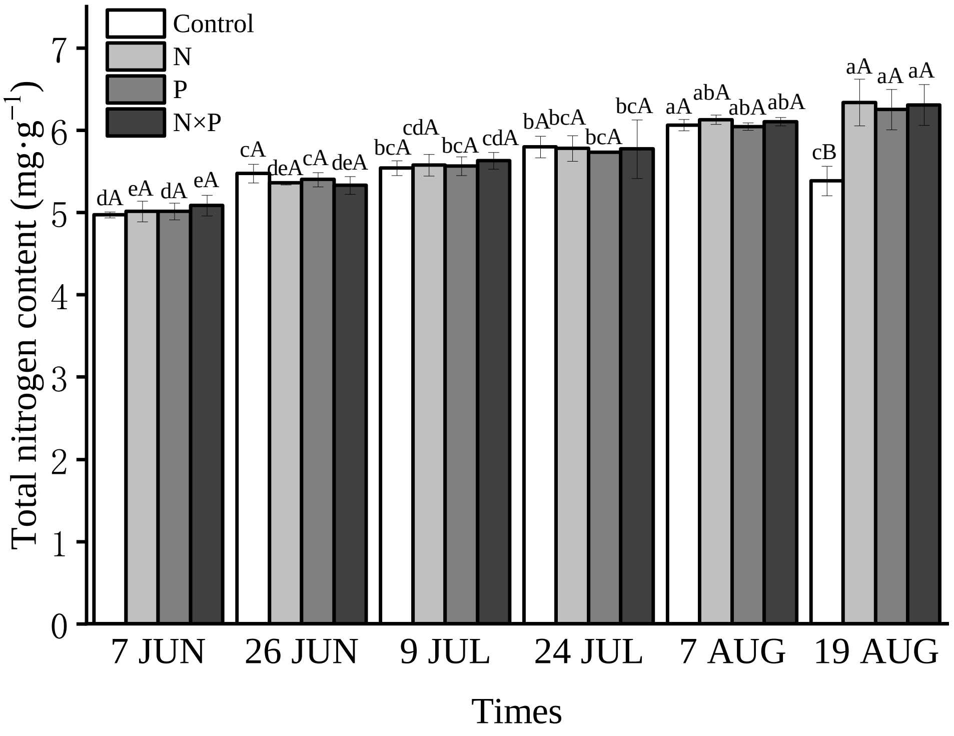

Figure S5 Seasonal variation of soil total nitrogen content under different nutrient additions

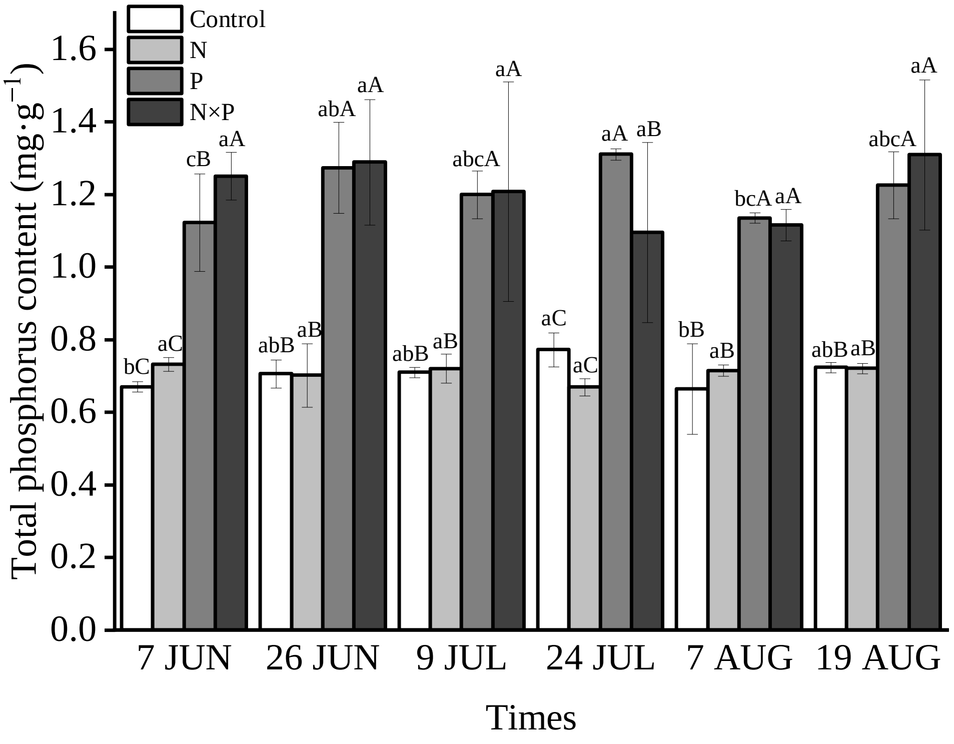

Figure S6 Seasonal variation of soil total phosphorus content under different nutrient additions

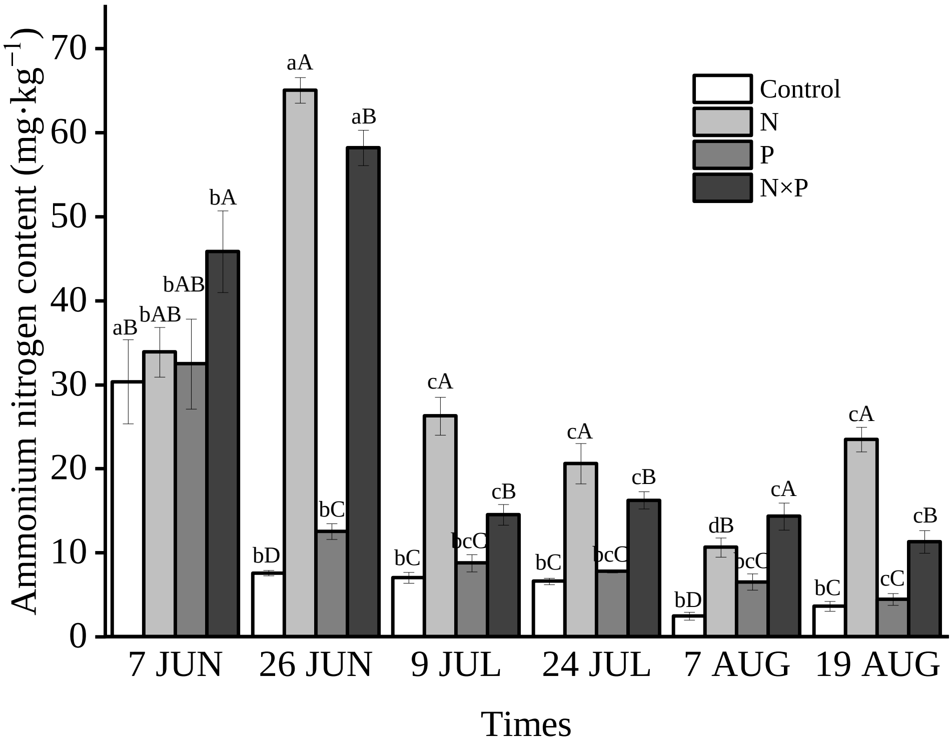

Figure S7 Seasonal changes in soil ammonium nitrogen content with different nutrient additions

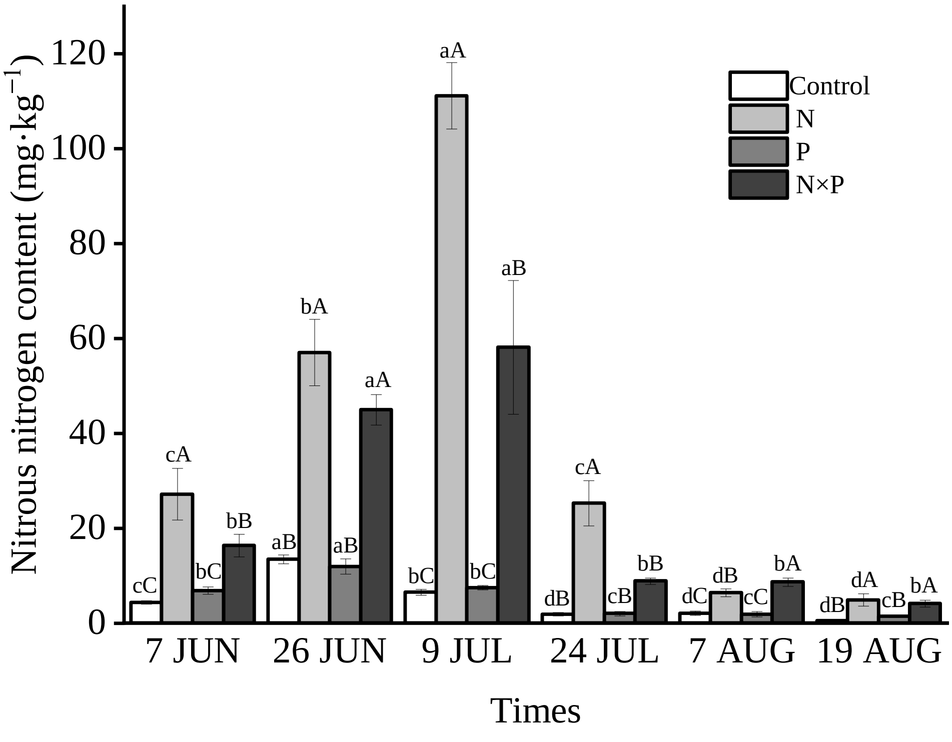

Figure S8 Seasonal variation of soil nitrate nitrogen content under different nutrient additions

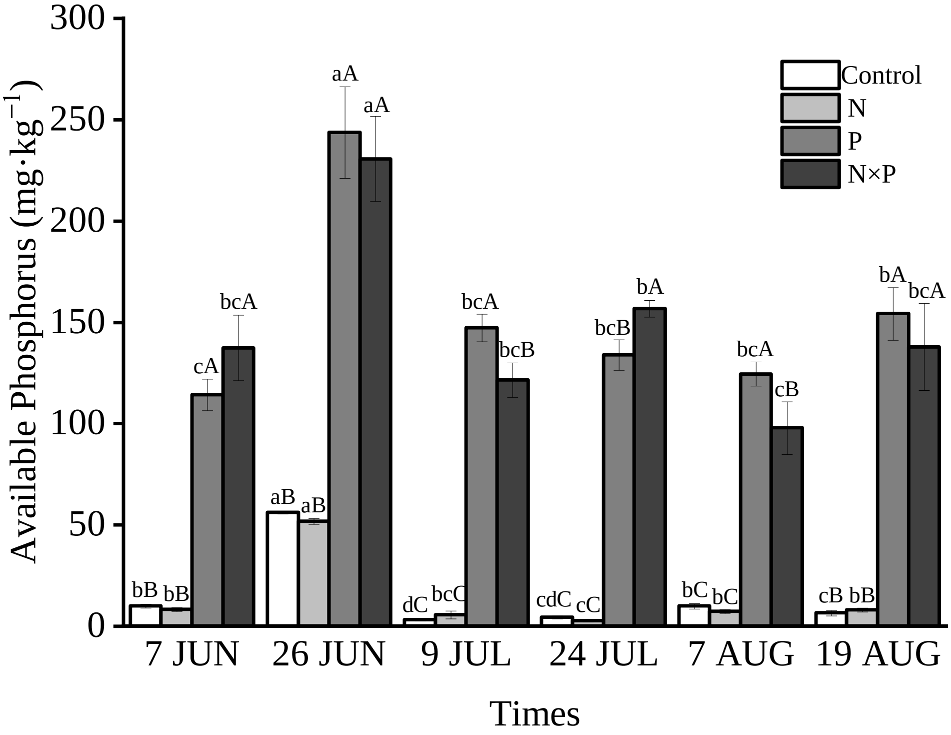

Figure S9 Seasonal variation of soil available phosphorus content under different nutrient additions

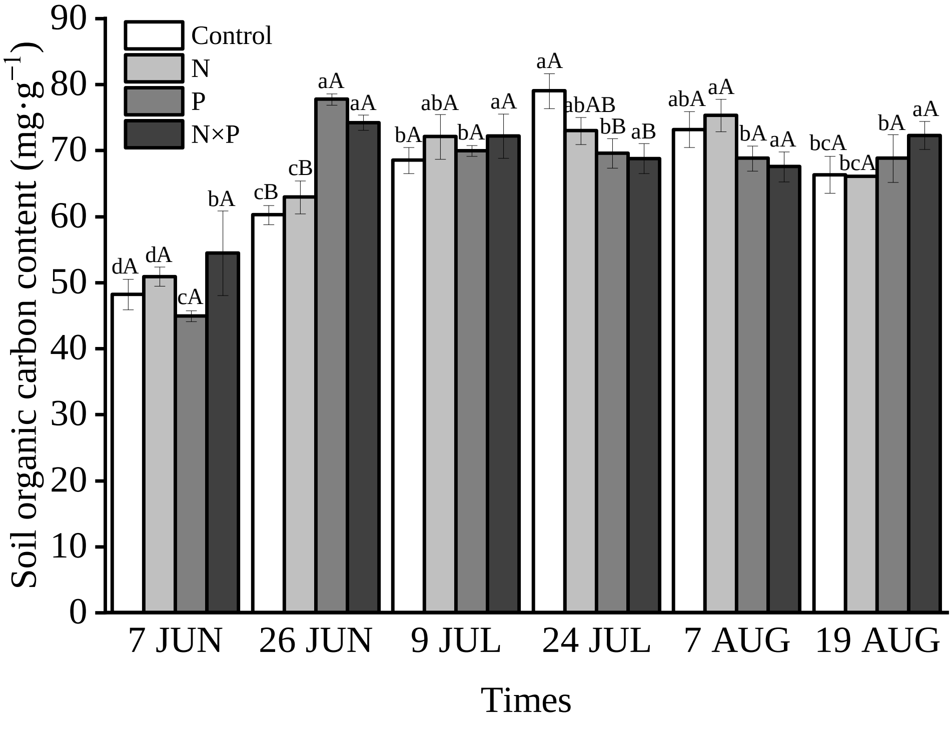

Figure S10 Seasonal variation of soil organic carbon content under different nutrient additions
