# Supplementary material for: Nutrient Additions Regulate Height Growth Rate but Not Biomass Growth Rate of Alpine Plants Through the Contrasting Effect of Total and Available Nitrogen
Source: Plants (Basel). 2025 Apr 6;14(7):1143. doi: 10.3390/plants14071143 (PMC11991464; doi:10.3390/plants14071143)
Supplement: Supplementary file 1 [file plants-14-01143-s001.zip › Table S1 Soil physical and chemical properties.pdf]

Table S1 Soil physical and chemical properties

Times	ST(℃)				SM（%）			
	Control	N	P	N×P	Control	N	P	N×P
7-Jun	8.73780	8.59683	8.34762	7.84821	28.01131	28.22371	25.91250	45.60982
7-Jun	8.55357	8.57798	8.20714	8.03274	31.79673	26.59762	20.50030	28.31339
7-Jun	8.96964	8.87083	8.36667	8.14643	27.67798	22.66756	27.15327	30.70774
7-Jun	8.75367	8.34167	8.55536	7.27024	29.16200	35.40595	32.13601	37.91875
26-Jun	13.17292	12.86617	12.60714	11.95149	16.63601	16.34772	15.88304	34.64851
26-Jun	13.05833	12.74494	12.40476	12.09315	26.01875	17.25298	10.77292	20.66161
26-Jun	13.13065	13.29464	12.54643	12.24077	13.62411	14.22024	15.56905	24.93690
26-Jun	13.12063	12.55893	13.09315	10.94226	18.75962	17.56994	17.47351	24.85833
9-Jul	14.59295	13.62489	13.60096	11.97853	17.27532	17.46688	18.28173	36.60897
9-Jul	14.43910	13.72692	13.91635	12.71987	25.62628	17.59840	13.51763	20.25417
9-Jul	14.54776	14.06506	13.72788	12.28974	16.91763	17.46891	14.05417	20.96763
9-Jul	14.52660	13.08269	14.21763	10.99135	19.93974	17.33333	24.03814	25.86090
24-Jul	14.09375	12.96766	13.19494	10.62470	16.48899	16.58671	16.55208	29.25744
24-Jul	14.34435	12.72083	13.73065	10.63185	21.19048	15.25923	14.54315	17.32768
24-Jul	13.90060	13.45476	13.24762	10.63125	16.31964	16.00298	15.02649	17.45982
24-Jul	14.11290	12.72738	13.64524	9.94970	17.99970	18.49792	23.90506	24.08720
7-Aug	15.41280	14.07381	13.75268	11.44702	6.05417	5.83373	5.97917	14.10744
7-Aug	15.13929	13.79405	14.41488	11.28720	7.03810	4.75565	5.71190	6.47440
7-Aug	15.43452	14.45536	14.12649	11.80625	4.98036	5.31577	4.52202	6.70655
7-Aug	15.32887	13.97202	14.54196	10.17619	6.02421	7.42976	9.71250	10.57708
19-Aug	15.83576	14.63877	14.27257	13.01910	4.87917	6.15984	7.39097	12.87014
19-Aug	15.29896	14.20938	14.76667	12.54757	4.07917	4.56250	7.48981	8.30174
19-Aug	15.99340	15.04340	14.56250	12.82882	4.66250	6.98646	4.98333	8.18194
19-Aug	15.70938	14.66354	14.66875	10.80417	4.54028	6.93056	10.92500	13.99514

Table S1 Soil physical and chemical properties

Times	pH				TN(mg·g^{-1})			
	Control	N	P	N×P	Control	N	P	N×P
7-Jun	7.17	6.51	6.99	6.54	4.91768	4.84327	4.88637	5.14929
7-Jun	6.67	6.59	6.59	6.26	4.98495	5.00675	4.97245	4.91518
7-Jun	6.84	6.46	6.69	6.36	4.99695	5.07265	5.08702	5.05893
7-Jun	6.55	6.47	7.17	6.43	4.98276	5.13504	5.09694	5.20701
26-Jun	6.94	6.68	6.64	6.64	5.41366	5.37273	5.39152	5.17580
26-Jun	7.29	6.65	6.62	6.57	5.57858	5.37264	5.51710	5.33931
26-Jun	6.95	6.39	6.58	6.30	5.55237	5.33078	5.30435	5.38497
26-Jun	6.95	6.96	6.73	6.57	5.34285	5.34802	5.37927	5.41908
9-Jul	6.93	6.36	6.5	6.37	5.41302	5.39793	5.45808	5.49778
9-Jul	6.81	6.44	6.59	6.23	5.53992	5.55675	5.49768	5.70805
9-Jul	6.84	6.48	6.63	6.32	5.57800	5.62093	5.58537	5.59217
9-Jul	6.71	6.48	6.61	6.37	5.61894	5.71069	5.71283	5.71099
24-Jul	7.05	6.72	6.59	6.49	5.78304	5.80862	5.71416	5.23525
24-Jul	6.87	6.75	6.55	6.6	5.85417	5.66797	5.73268	5.90651
24-Jul	6.8	6.59	6.5	6.34	5.62054	5.98070	5.74451	5.93932
24-Jul	6.83	6.54	6.82	6.52	5.93051	5.64330	5.72874	5.99390
7-Aug	6.81	6.39	7.21	6.53	5.95112	5.98151	5.95481	5.97960
7-Aug	6.87	6.64	6.87	6.63	5.97761	6.10086	6.00150	6.12068
7-Aug	7.51	6.77	6.43	6.71	6.06828	6.25812	6.16992	6.08176
7-Aug	6.87	6.48	6.95	6.52	6.24888	6.16248	6.04208	6.23165
19-Aug	6.76	6.56	6.61	6.34	4.85392	5.48121	5.52170	5.66308
19-Aug	7.31	6.66	6.46	6.66	5.52170	6.55875	6.48225	6.64583
19-Aug	6.64	6.49	6.36	6.39	5.48121	6.63296	6.59505	6.17008
19-Aug	6.93	6.37	6.38	6.33	5.66308	6.67078	6.39305	6.74498

Table S1 Soil physical and chemical properties

Times	TP(mg·g^{-1})				NH_4^+-N(mg·kg^{-1})			
	Control	N	P	N×P	Control	N	P	N×P
7-Jun	0.68338	0.73183	0.98860	1.31460	34.48639	33.88184	36.23636	47.74590
7-Jun	0.67024	0.70519	1.27867	1.27598	21.76017	30.36831	44.79134	56.71575
7-Jun	0.67619	0.74091	1.18573	1.16068	42.55790	42.23902	19.46010	33.12244
7-Jun	0.65116	0.74880	1.03653	1.25090	22.61328	29.03819	29.40394	45.86137
26-Jun	0.66707	0.80768	1.29576	1.28880	8.01421	62.15775	12.52499	53.70103
26-Jun	0.69105	0.72760	1.43040	1.53078	6.63354	62.65930	14.35497	57.13759
26-Jun	0.70567	0.60419	1.23640	1.19664	7.57096	68.01958	9.89550	58.19891
26-Jun	0.75888	0.66680	1.13014	1.13898	8.06512	67.39521	13.32449	63.75810
9-Jul	0.73000	0.68930	1.17819	0.81996	6.04301	30.14794	6.04254	11.47730
9-Jul	0.71092	0.74680	1.29461	1.55880	7.01544	19.96665	11.02891	14.48913
9-Jul	0.70076	0.76170	1.14269	1.20796	8.79743	28.69150	8.48137	14.50638
9-Jul	0.69888	0.68280	1.18225	1.24511	6.20587	26.26869	9.47480	17.55272
24-Jul	0.82381	0.70264	1.30232	1.23381	6.60201	21.90585	8.05683	18.31085
24-Jul	0.78244	0.65600	1.29701	0.74720	6.73637	25.67084	7.77745	17.43546
24-Jul	0.77208	0.66925	1.31053	1.09532	7.47921	20.60640	7.89685	13.75909
24-Jul	0.71000	0.64910	1.33227	1.30496	5.59043	14.24253	7.37867	15.37594
7-Aug	0.73631	0.70712	1.15088	1.07820	1.35500	13.58810	6.52087	11.48570
7-Aug	0.48381	0.71505	1.13515	1.13119	2.46159	11.32510	4.29453	14.31677
7-Aug	0.75830	0.73662	1.13878	1.16980	2.47217	9.08733	6.22418	12.69412
7-Aug	0.68020	0.70140	1.11580	1.08213	3.55759	8.52469	9.04389	18.77048
19-Aug	0.72992	0.71594	1.22200	1.33573	3.24408	21.86975	3.17820	13.71241
19-Aug	0.73680	0.70663	1.34012	1.54810	3.62370	26.83419	6.47577	13.34700
19-Aug	0.70379	0.73941	1.11502	1.04391	5.24193	24.92723	3.69213	8.18863
19-Aug	0.72351	0.72066	1.22571	1.30925	2.38509	20.29447	4.44870	9.99806

Table S1 Soil physical and chemical properties

Times	NO_3^--N($mg\cdot kg^{-1}$)				AP($mg\cdot kg^{-1}$)			
	Control	N	P	N×P	Control	N	P	N×P
7-Jun	3.28203	24.79014	5.05513	22.49474	9.76906	5.83137	109.77633	162.98094
7-Jun	4.71924	29.01704	6.95513	15.91892	12.97066	10.58111	114.23699	143.84669
7-Jun	4.89445	40.65820	8.71444	16.36328	8.32118	7.81437	135.07948	90.21926
7-Jun	4.53782	14.32860	6.84018	10.67619	8.74545	8.60606	97.85517	152.21516
26-Jun	15.29608	69.52432	11.62145	35.99772	57.76884	48.05267	228.08017	199.23490
26-Jun	14.22971	37.53856	15.98698	45.02138	56.53668	55.29475	308.30051	291.22372
26-Jun	13.35830	57.06762	8.30149	50.01154	56.18433	51.64710	235.48516	226.72304
26-Jun	11.04464	64.13997	11.96997	49.05488	54.24745	51.59388	202.89565	205.21791
9-Jul	7.75358	111.17677	7.51420	44.94581	2.67775	1.26949	138.02437	124.49819
9-Jul	6.17920	111.17677	6.54793	32.19975	4.69474	3.52535	137.13949	144.11236
9-Jul	7.21507	94.08443	7.32253	97.29838	1.73539	9.37837	147.13795	106.65307
9-Jul	4.84505	128.26911	8.67215	58.14798	3.98000	8.47000	166.25000	110.46000
24-Jul	1.91044	25.30408	0.92871	7.84340	5.38392	3.40982	135.92209	150.09781
24-Jul	1.81835	36.65949	2.03070	8.04975	5.56233	2.58749	147.91962	157.68923
24-Jul	1.03377	13.14048	1.99475	8.86578	4.43281	2.08184	112.45030	150.65025
24-Jul	2.87920	26.11226	3.16863	10.70419	2.35218	2.27082	139.30554	167.98950
7-Aug	2.10806	4.67892	0.89170	8.77358	13.04938	6.69304	127.19211	59.57995
7-Aug	3.32157	5.59853	1.87633	10.76131	11.33094	9.89703	137.33203	118.38632
7-Aug	1.18601	8.39156	1.25016	8.66620	7.84592	7.60209	108.76695	107.56761
7-Aug	1.81660	7.23286	3.48713	6.46370	7.30971	5.06291	124.43036	105.72370
19-Aug	0.90372	2.12451	1.40877	4.72451	6.44147	11.05709	152.06160	137.70064
19-Aug	0.55221	6.63385	1.05949	5.81059	6.12378	6.36444	182.56829	185.35043
19-Aug	0.67802	7.56366	1.20958	2.41077	3.60197	6.79549	120.22363	81.24975
19-Aug	0.11261	3.29838	1.95725	3.57500	9.59866	7.73407	161.83761	146.96277

Table S1 Soil physical and chemical properties

Times	SOC(mg·g^{-1})			
	Control	N	P	N×P
7-Jun	41.36213	54.85372	44.91018	43.35548
7-Jun	51.32890	50.89587	44.95097	68.77076
7-Jun	50.33223	49.83389	42.94274	61.83511
7-Jun	49.93342	48.00000	47.00000	43.88298
26-Jun	63.50000	69.50000	75.50000	71.30984
26-Jun	56.38723	57.46169	77.89614	74.23577
26-Jun	60.24166	61.79402	79.84032	74.50000
26-Jun	60.83777	62.91857	77.74549	76.89747
9-Jul	71.60679	71.51163	69.95383	80.53524
9-Jul	70.95270	62.82392	67.90945	71.76080
9-Jul	68.50584	78.00000	71.95203	72.19392
9-Jul	62.95803	76.00000	70.00000	64.28571
24-Jul	82.75000	75.59880	63.62275	62.33378
24-Jul	79.01722	68.56715	74.50166	72.00997
24-Jul	82.69487	70.60878	70.60878	68.78457
24-Jul	71.60679	77.25000	69.57773	72.00997
7-Aug	71.80851	76.29654	68.81850	67.55675
7-Aug	78.73754	68.81649	69.81383	66.00000
7-Aug	66.27907	75.29920	72.85429	73.75415
7-Aug	75.89880	80.78457	63.78738	62.91611
19-Aug	61.91744	65.95603	64.28571	66.41145
19-Aug	64.00000	65.86826	60.95936	72.30326
19-Aug	74.50000	66.32314	75.00000	74.25249
19-Aug	64.87026	66.04914	75.00000	76.24585
