# Supplementary material for: Nutrient Additions Regulate Height Growth Rate but Not Biomass Growth Rate of Alpine Plants Through the Contrasting Effect of Total and Available Nitrogen
Source: Plants (Basel). 2025 Apr 6;14(7):1143. doi: 10.3390/plants14071143 (PMC11991464; doi:10.3390/plants14071143)
Supplement: Supplementary file 1 [file plants-14-01143-s001.zip › Table S2 Relative growth rate.pdf]

Table S2 Relative growth rate

Treatment	Species	RGR_B ($g \cdot g^{-1} \cdot d^{-1}$)	RGR_H ($cm \cdot cm^{-1} \cdot d^{-1}$)	seasonal dynamics RGR_H($cm \cdot cm^{-1} \cdot d^{-1}$)				
				7JUN-26JUN	26JUN-9JUL	9JUL-24JUL	24JUL-7AUG	7AUG-19AUG
Control	*P. versicolor*	0.04042	0.01997	0.01878	0.03371	0.03603	0.01575	0.00171
Control	*A. obtusiloba*	0.04740	0.02735	0.04486	0.06719	0.02965	0.00572	0.00098
Control	*R. membranaceus*	0.05144	0.01801	0.03545	0.02919	0.02017	0.01003	0.00188
Control	*M. chinensis*	0.05850	0.02338	0.03260	0.03702	0.02540	0.02590	0.00332
Control	*T. lanceolata*	0.08226	0.01478	0.04360	0.02503	0.01058	0.00127	0.00033
Control	*E. nutans*	0.05203	0.02183	0.02666	0.03093	0.03242	0.01931	0.00393
Control	*P. saundersiana*	0.04820	0.02002	0.03036	0.04902	0.01931	0.00883	0.00085
Control	*M. ruthenica*	0.05179	0.02044	0.03119	0.04957	0.02403	0.00493	0.00092
Control	*S. nigrescens*	0.03762	0.01368	0.01933	0.02213	0.01712	0.00343	0.00881
Control	*G. straminea*	0.04825	0.01937	0.03615	0.03225	0.01882	0.00776	0.00429
Control	*A. diplostephioides*	0.05029	0.01393	0.02882	0.02565	0.01615	0.00251	0.00188
Control	*G. boreale*	0.06537	0.01585	0.04129	0.01787	0.01901	0.00377	0.00142
Control	*S. pulchra*	0.03003	0.01245	0.02151	0.02800	0.01322	0.00699	0.00457
Control	*O. kansuensis*	0.05673	0.02327	0.02703	0.04264	0.03194	0.02091	0.00167
N×P	*P. versicolor*	0.04817	0.03110	0.04716	0.04767	0.04174	0.02479	0.00416
N×P	*A. obtusiloba*	0.05546	0.02963	0.04098	0.06349	0.01072	0.04372	0.00074
N×P	*R. membranaceus*	0.04012	0.02426	0.04605	0.05737	0.01383	0.01467	0.00020
N×P	*M. chinensis*	0.07398	0.02713	0.03719	0.07100	0.02590	0.01011	0.00239
N×P	*T. lanceolata*	0.06406	0.02831	0.08056	0.04514	0.02176	0.00639	0.00055
N×P	*E. nutans*	0.05101	0.02955	0.03710	0.05497	0.03395	0.02640	0.00377
N×P	*P. saundersiana*	0.03848	0.02729	0.04247	0.05505	0.04487	0.00411	0.00062
N×P	*M. ruthenica*	0.05088	0.03236	0.03929	0.07251	0.04321	0.01823	0.00091
N×P	*S. nigrescens*	0.04392	0.03116	0.06518	0.06495	0.03420	0.00378	0.00113
N×P	*G. straminea*	0.04531	0.02127	0.04963	0.04104	0.01427	0.00947	0.00114
N×P	*A. diplostephioides*	0.05784	0.02494	0.05888	0.05332	0.02168	0.00211	0.00023
N×P	*G. boreale*	0.05618	0.02880	0.06142	0.05484	0.02966	0.00910	0.00110
N×P	*S. pulchra*	0.01681	0.01820	0.02403	0.03892	0.03304	0.00449	0.00016

N×P	*O. kansuensis*	0.02717	0.02512	0.03929	0.05908	0.02255	0.01376	0.00119
N×P	*P. versicolor*	0.02067	0.02757	0.04243	0.05239	0.03655	0.01311	0.00216
N×P	*A. obtusiloba*	0.04266	0.02344	0.04305	0.04688	0.02826	0.00836	0.00032
N×P	*R. membranaceus*	0.03064	0.02196	0.03160	0.05482	0.03063	0.00178	0.00023
N×P	*M. chinensis*	0.07755	0.02374	0.03735	0.04780	0.03261	0.00912	0.00101
N×P	*T. lanceolata*	0.07130	0.02559	0.06326	0.03498	0.02987	0.00891	0.00123
N×P	*E. nutans*	0.04339	0.03005	0.03603	0.06019	0.03643	0.02321	0.00431
N×P	*P. saundersiana*	0.05016	0.02130	0.04082	0.03366	0.03045	0.00782	0.00162
N×P	*M. ruthenica*	0.04775	0.03146	0.04699	0.05549	0.06009	0.00456	0.00154
N×P	*S. nigrescens*	0.04379	0.02648	0.05030	0.05634	0.02850	0.00447	0.00327
N×P	*G. straminea*	0.04408	0.01949	0.02938	0.04002	0.01832	0.01457	0.00227
N×P	*A. diplostephioides*	0.06112	0.02422	0.04963	0.05519	0.02238	0.00445	0.00030
N×P	*G. boreale*	0.07384	0.03016	0.06602	0.05163	0.03053	0.00989	0.00422
N×P	*S. pulchra*	0.04103	0.02161	0.01969	0.05695	0.02859	0.00767	0.00288
N×P	*O. kansuensis*	0.00923	0.02573	0.04305	0.04908	0.02188	0.01790	0.00543
Control	*P. versicolor*	0.02327	0.02476	0.05127	0.04904	0.02181	0.00916	0.00251
Control	*A. obtusiloba*	0.05024	0.02754	0.06673	0.05634	0.01256	0.01240	0.00211
Control	*R. membranaceus*	0.03955	0.01601	0.03615	0.02829	0.01022	0.01101	0.00201
Control	*M. chinensis*	0.07404	0.02113	0.04808	0.03368	0.02450	0.00743	0.00064
Control	*T. lanceolata*	0.02505	0.01831	0.04728	0.02521	0.02042	0.00569	0.00056
Control	*E. nutans*	0.04764	0.02200	0.01812	0.05236	0.02144	0.01828	0.00621
Control	*P. saundersiana*	0.05610	0.02439	0.04835	0.06805	0.01685	0.00052	0.00026
Control	*M. ruthenica*	0.03328	0.02214	0.04047	0.04404	0.03311	0.01092	0.00103
Control	*S. nigrescens*	0.03932	0.01728	0.04108	0.03465	0.00954	0.00203	0.00544
Control	*G. straminea*	0.04347	0.01908	0.04414	0.03155	0.01168	0.00904	0.00554
Control	*A. diplostephioides*	0.04908	0.01615	0.03615	0.03119	0.01534	0.00865	0.00405
Control	*G. boreale*	0.06149	0.01536	0.03288	0.01694	0.02260	0.00296	0.00558
Control	*S. pulchra*	0.03343	0.01135	0.02156	0.01682	0.01594	0.00300	0.00296
Control	*O. kansuensis*	0.05695	0.01996	0.02744	0.04275	0.02380	0.01269	0.00077
P	*P. versicolor*	0.03809	0.02788	0.04345	0.04830	0.04190	0.01376	0.00197

P	*A. obtusiloba*	0.04803	0.03025	0.04492	0.08882	0.02794	0.00287	0.00044
P	*R. membranaceus*	0.02585	0.01948	0.03647	0.05133	0.01720	0.00117	0.00022
P	*M. chinensis*	0.08668	0.02849	0.04663	0.05448	0.04103	0.01058	0.00080
P	*T. lanceolata*	0.09457	0.02160	0.06025	0.02940	0.02221	0.00448	0.00089
P	*E. nutans*	0.05112	0.02736	0.03909	0.03845	0.05118	0.01527	0.00174
P	*P. saundersiana*	0.06767	0.01554	0.02792	0.04447	0.00665	0.00386	0.00173
P	*M. ruthenica*	0.06216	0.03227	0.04460	0.07468	0.04028	0.01083	0.00320
P	*S. nigrescens*	0.04246	0.02213	0.04856	0.03995	0.02620	0.00186	0.00278
P	*G. straminea*	0.04163	0.01975	0.02565	0.05099	0.02245	0.00578	0.00170
P	*A. diplostephioides*	0.04233	0.02053	0.02714	0.05227	0.02528	0.00401	0.00198
P	*G. boreale*	0.05496	0.01659	0.04489	0.02413	0.01616	0.00300	0.00161
P	*S. pulchra*	0.02812	0.01794	0.02588	0.03752	0.02854	0.00328	0.00123
P	*O. kansuensis*	0.04686	0.02188	0.01402	0.05748	0.02684	0.01637	0.00228
N	*P. versicolor*	0.02939	0.02454	0.04115	0.05549	0.02055	0.01290	0.00236
N	*A. obtusiloba*	0.03466	0.02313	0.05183	0.03169	0.02417	0.01527	0.00154
N	*R. membranaceus*	0.03894	0.02078	0.03389	0.01663	0.04401	0.01413	0.00141
N	*M. chinensis*	0.07001	0.02360	0.03281	0.05224	0.03256	0.00898	0.00065
N	*T. lanceolata*	0.09140	0.01811	0.06102	0.01981	0.01389	0.00318	0.00080
N	*E. nutans*	0.04785	0.01768	0.01933	0.04801	0.00954	0.01444	0.00344
N	*P. saundersiana*	0.07164	0.01961	0.03514	0.04521	0.02153	0.00335	0.00108
N	*M. ruthenica*	0.04361	0.02413	0.04699	0.04643	0.02193	0.01206	0.00263
N	*S. nigrescens*	0.03466	0.01868	0.03677	0.02478	0.02722	0.01050	0.00094
N	*G. straminea*	0.04713	0.02047	0.04224	0.03710	0.01354	0.01050	0.00589
N	*A. diplostephioides*	0.05500	0.01661	0.03669	0.03648	0.01313	0.00373	0.00047
N	*G. boreale*	0.06534	0.01576	0.05042	0.01950	0.01086	0.00136	0.00306
N	*S. pulchra*	0.03011	0.00979	0.00700	0.02044	0.01782	0.00183	0.00407
N	*O. kansuensis*	0.03857	0.02290	0.04879	0.03424	0.02492	0.01335	0.00186
N	*P. versicolor*	0.02837	0.02270	0.02783	0.05863	0.01965	0.01280	0.00314
N	*A. obtusiloba*	0.08020	0.01623	0.02540	0.04706	0.01221	0.00300	0.00076
N	*R. membranaceus*	0.03693	0.01351	0.02588	0.02394	0.00860	0.01339	0.00094

N	*M. chinensis*	0.08084	0.02473	0.03999	0.05486	0.02656	0.01171	0.00064
N	*T. lanceolata*	0.07903	0.01730	0.03852	0.03273	0.01692	0.00537	0.00040
N	*E. nutans*	0.04450	0.02574	0.03119	0.04923	0.02706	0.02583	0.00373
N	*P. saundersiana*	0.07757	0.01726	0.03119	0.03119	0.01245	0.01688	0.00228
N	*M. ruthenica*	0.03638	0.02455	0.03477	0.03788	0.03997	0.01433	0.00550
N	*S. nigrescens*	0.05075	0.01703	0.03349	0.02491	0.02532	0.00514	0.00227
N	*G. straminea*	0.04130	0.01916	0.03566	0.02952	0.01856	0.01322	0.00687
N	*A. diplostephioides*	0.05175	0.01618	0.03549	0.02777	0.01891	0.00245	0.00244
N	*G. boreale*	0.05562	0.01934	0.04025	0.04098	0.01957	0.00782	0.00137
N	*S. pulchra*	0.02388	0.01084	0.02213	0.01432	0.01987	0.00201	0.00112
N	*O. kansuensis*	0.04842	0.02216	0.04492	0.03504	0.02353	0.01101	0.00403
P	*P. versicolor*	0.03313	0.02875	0.03119	0.06463	0.04445	0.01050	0.00306
P	*A. obtusiloba*	0.05079	0.02545	0.04172	0.06867	0.02428	0.00179	0.00181
P	*R. membranaceus*	0.02431	0.02751	0.06130	0.05754	0.02670	0.00383	0.00048
P	*M. chinensis*	0.06898	0.02793	0.04575	0.05265	0.04233	0.00902	0.00070
P	*T. lanceolata*	0.06055	0.02142	0.04257	0.03371	0.02858	0.00973	0.00078
P	*E. nutans*	0.04702	0.03031	0.04521	0.04740	0.04394	0.02122	0.00367
P	*P. saundersiana*	0.07477	0.02397	0.04357	0.05227	0.03121	0.00187	0.00095
P	*M. ruthenica*	0.04695	0.03198	0.03788	0.06876	0.04869	0.01411	0.00206
P	*S. nigrescens*	0.02539	0.02478	0.04447	0.02260	0.04412	0.01733	0.00291
P	*G. straminea*	0.04007	0.02077	0.04163	0.03308	0.02089	0.01101	0.00431
P	*A. diplostephioides*	0.05146	0.02033	0.04549	0.03819	0.01898	0.00461	0.00254
P	*G. boreale*	0.06368	0.02407	0.05127	0.04275	0.02380	0.01033	0.00184
P	*S. pulchra*	0.02844	0.01401	0.02336	0.02614	0.01994	0.00270	0.00268
P	*O. kansuensis*	0.02693	0.02534	0.05203	0.03981	0.02475	0.01715	0.00244
Control	*P. versicolor*	0.03586	0.02356	0.01855	0.04908	0.03458	0.01950	0.00334
Control	*A. obtusiloba*	0.05976	0.02881	0.05332	0.06989	0.02878	0.00326	0.00136
Control	*R. membranaceus*	0.03102	0.01610	0.02858	0.02849	0.02622	0.00146	0.00166
Control	*M. chinensis*	0.07758	0.02838	0.03477	0.05872	0.04219	0.01452	0.00188
Control	*T. lanceolata*	0.05363	0.01632	0.03657	0.03049	0.01302	0.00720	0.00113

Control	*E. nutans*	0.04830	0.02716	0.03525	0.04925	0.04068	0.01594	0.00359
Control	*P. saundersiana*	0.06453	0.02084	0.02849	0.05240	0.02452	0.00342	0.00086
Control	*M. ruthenica*	0.05067	0.02277	0.03314	0.04082	0.04406	0.00186	0.00431
Control	*S. nigrescens*	0.03852	0.02316	0.04937	0.03889	0.01433	0.02078	0.00160
Control	*G. straminea*	0.04751	0.01769	0.03119	0.03535	0.01787	0.01034	0.00078
Control	*A. diplostephioides*	0.04360	0.01559	0.03819	0.02296	0.01548	0.00706	0.00065
Control	*G. boreale*	0.05806	0.01761	0.04808	0.02711	0.01111	0.00354	0.00472
Control	*S. pulchra*	0.03051	0.01345	0.02946	0.00828	0.02445	0.00500	0.00363
Control	*O. kansuensis*	0.04015	0.02243	0.03514	0.04157	0.03235	0.00987	0.00147
N×P	*P. versicolor*	0.03008	0.03133	0.05244	0.05332	0.04148	0.01798	0.00271
N×P	*A. obtusiloba*	0.05340	0.02632	0.04305	0.07739	0.01486	0.00769	0.00075
N×P	*R. membranaceus*	0.02518	0.01900	0.02549	0.05705	0.01903	0.00124	0.00063
N×P	*M. chinensis*	0.06880	0.02177	0.04927	0.03783	0.02757	0.00314	0.00030
N×P	*T. lanceolata*	0.07625	0.02011	0.05082	0.02919	0.01837	0.01006	0.00056
N×P	*E. nutans*	0.04396	0.03087	0.03780	0.05599	0.03616	0.02912	0.00496
N×P	*P. saundersiana*	0.06214	0.02815	0.03838	0.05602	0.05043	0.00583	0.00065
N×P	*M. ruthenica*	0.05670	0.03422	0.03735	0.07728	0.04097	0.02346	0.00398
N×P	*S. nigrescens*	0.03510	0.02292	0.02902	0.05655	0.03246	0.00244	0.00273
N×P	*G. straminea*	0.04679	0.01784	0.03999	0.03783	0.01487	0.00282	0.00142
N×P	*A. diplostephioides*	0.05194	0.01999	0.03735	0.02817	0.02928	0.01164	0.00083
N×P	*G. boreale*	0.07091	0.02220	0.05686	0.03804	0.01734	0.00505	0.00283
N×P	*S. pulchra*	0.03475	0.01526	0.02161	0.03066	0.02030	0.00767	0.00152
N×P	*O. kansuensis*	0.02719	0.02144	0.03119	0.04789	0.03068	0.00546	0.00054
P	*P. versicolor*	0.04225	0.02499	0.03296	0.05390	0.02806	0.01587	0.00308
P	*A. obtusiloba*	0.04281	0.02515	0.05478	0.07689	0.00386	0.00231	0.00070
P	*R. membranaceus*	0.02310	0.02164	0.03984	0.05960	0.01538	0.00304	0.00042
P	*M. chinensis*	0.07054	0.02582	0.04778	0.03814	0.03866	0.01371	0.00048
P	*T. lanceolata*	0.11433	0.02157	0.06315	0.02588	0.02330	0.00429	0.00054
P	*E. nutans*	0.04143	0.02325	0.03273	0.03295	0.04325	0.01771	0.00193
P	*P. saundersiana*	0.05513	0.02429	0.03399	0.05052	0.03282	0.00855	0.00163

P	*M. ruthenica*	0.04215	0.02908	0.03838	0.05863	0.03130	0.01719	0.00418
P	*S. nigrescens*	0.04228	0.02849	0.06758	0.04521	0.03575	0.00500	0.00080
P	*G. straminea*	0.04909	0.01916	0.03253	0.05463	0.01618	0.00486	0.00111
P	*A. diplostephioides*	0.05896	0.01584	0.03492	0.03119	0.01709	0.00563	0.00232
P	*G. boreale*	0.05334	0.01895	0.05163	0.03288	0.01145	0.00510	0.00188
P	*S. pulchra*	0.03487	0.01405	0.02092	0.02996	0.02420	0.00134	0.00193
P	*O. kansuensis*	0.05205	0.02164	0.03894	0.04199	0.02223	0.01288	0.00080
Control	*P. versicolor*	0.04032	0.01865	0.01679	0.03559	0.02251	0.02006	0.00366
Control	*A. obtusiloba*	0.04139	0.02650	0.05163	0.07480	0.01606	0.00236	0.00036
Control	*R. membranaceus*	0.03038	0.01851	0.04381	0.04247	0.00107	0.01311	0.00069
Control	*M. chinensis*	0.07395	0.01603	0.03778	0.03233	0.01337	0.00395	0.00157
Control	*T. lanceolata*	0.10287	0.01719	0.02494	0.01906	0.01026	0.00852	0.00069
Control	*E. nutans*	0.03488	0.01940	0.03576	0.04875	0.01870	0.00148	0.00085
Control	*P. saundersiana*	0.05596	0.02604	0.03990	0.03002	0.02245	0.03337	0.00304
Control	*M. ruthenica*	0.04300	0.02489	0.03584	0.03363	0.03092	0.00733	0.00517
Control	*S. nigrescens*	0.03414	0.00145	0.03553	0.03230	0.01043	0.00510	0.00250
Control	*G. straminea*	0.04499	0.01935	0.04147	0.03041	0.01852	0.00383	0.00580
Control	*A. diplostephioides*	0.03944	0.01312	0.02945	0.01834	0.01374	0.00517	0.00253
Control	*G. boreale*	0.06210	0.01346	0.02588	0.02386	0.01415	0.00274	0.00480
Control	*S. pulchra*	0.03106	0.01324	0.02623	0.01952	0.01740	0.00646	0.00136
Control	*O. kansuensis*	0.04521	0.01948	0.03852	0.04159	0.01874	0.00619	0.00069
P	*P. versicolor*	0.03587	0.02688	0.03580	0.06454	0.02690	0.01505	0.00243
P	*A. obtusiloba*	0.03264	0.02740	0.06172	0.06384	0.02396	0.00569	0.00030
P	*R. membranaceus*	0.03872	0.02051	0.03899	0.04344	0.02030	0.00800	0.00051
P	*M. chinensis*	0.06082	0.02382	0.04663	0.04204	0.03497	0.00447	0.00056
P	*T. lanceolata*	0.07913	0.02235	0.02518	0.02233	0.02465	0.00537	0.00053
P	*E. nutans*	0.05040	0.02803	0.02048	0.05769	0.04592	0.01566	0.00335
P	*P. saundersiana*	0.07075	0.02542	0.03615	0.05332	0.02946	0.01740	0.00060
P	*M. ruthenica*	0.05622	0.02621	0.01402	0.06094	0.03117	0.03011	0.00303
P	*S. nigrescens*	0.02696	0.02591	0.04491	0.05093	0.02921	0.00389	0.00145

P	*G. straminea*	0.04961	0.00359	0.03390	0.03952	0.01387	0.00700	0.00289
P	*A. diplostephioides*	0.04420	0.01427	0.02325	0.03119	0.02284	0.00148	0.00118
P	*G. boreale*	0.06329	0.01843	0.01663	0.02744	0.01026	0.00489	0.00845
P	*S. pulchra*	0.02838	0.01633	0.02079	0.04247	0.01973	0.00476	0.00059
P	*O. kansuensis*	0.04723	0.02343	0.02375	0.04441	0.03943	0.01666	0.00088
N	*P. versicolor*	0.03873	0.02905	0.04562	0.04589	0.03680	0.02395	0.00285
N	*A. obtusiloba*	0.04629	0.02491	0.05602	0.04782	0.01527	0.00572	0.00055
N	*R. membranaceus*	0.04122	0.01704	0.03653	0.03119	0.02269	0.00167	0.00032
N	*M. chinensis*	0.07037	0.01669	0.03230	0.03752	0.01155	0.00853	0.00073
N	*T. lanceolata*	0.06793	0.01492	0.05123	0.02034	0.01029	0.00912	0.00068
N	*E. nutans*	0.03989	0.02449	0.04587	0.03417	0.03975	0.00799	0.00218
N	*P. saundersiana*	0.06281	0.02419	0.03615	0.05170	0.03096	0.01136	0.00041
N	*M. ruthenica*	0.03731	0.02368	0.01402	0.05491	0.03935	0.01842	0.00156
N	*S. nigrescens*	0.04460	0.01951	0.05748	0.02413	0.02258	0.00198	0.00084
N	*G. straminea*	0.04185	0.01910	0.04982	0.02433	0.01373	0.01360	0.00158
N	*A. diplostephioides*	0.03216	0.01718	0.03213	0.03425	0.02110	0.00245	0.00244
N	*G. boreale*	0.06193	0.01456	0.04178	0.01990	0.01010	0.00625	0.00097
N	*S. pulchra*	0.02474	0.01287	0.01301	0.03288	0.01898	0.00157	0.00236
N	*O. kansuensis*	0.04937	0.01836	0.02902	0.04146	0.02144	0.00174	0.00293
N	*P. versicolor*	0.03663	0.02380	0.03735	0.03466	0.03856	0.01336	0.00282
N	*A. obtusiloba*	0.03685	0.01836	0.05332	0.03959	0.00703	0.00225	0.00144
N	*R. membranaceus*	0.03013	0.01148	0.02074	0.00922	0.02084	0.00735	0.00236
N	*M. chinensis*	0.07757	0.02502	0.03615	0.05380	0.03666	0.00731	0.00092
N	*T. lanceolata*	0.06359	0.01489	0.04766	0.02283	0.00905	0.00168	0.00032
N	*E. nutans*	0.03709	0.02146	0.02537	0.03032	0.03088	0.01723	0.00585
N	*P. saundersiana*	0.06544	0.02128	0.03858	0.04828	0.02622	0.00147	0.00084
N	*M. ruthenica*	0.02933	0.02529	0.03559	0.03580	0.05355	0.00456	0.00440
N	*S. nigrescens*	0.03640	0.01494	0.03207	0.02125	0.02297	0.00089	0.00268
N	*G. straminea*	0.04173	0.01805	0.04426	0.02724	0.01416	0.01005	0.00171
N	*A. diplostephioides*	0.03747	0.02089	0.02588	0.02934	0.04685	0.00931	0.00257

N	*G. boreale*	0.06699	0.01563	0.04762	0.02380	0.01118	0.00098	0.00149
N	*S. pulchra*	0.02630	0.01122	0.01840	0.01878	0.01782	0.00362	0.00136
N	*O. kansuensis*	0.03164	0.02198	0.04709	0.01954	0.03555	0.00622	0.00474
N×P	*P. versicolor*	0.03209	0.03198	0.05427	0.05236	0.04793	0.01497	0.00198
N×P	*A. obtusiloba*	0.06005	0.02757	0.05569	0.05948	0.03077	0.00349	0.00048
N×P	*R. membranaceus*	0.03417	0.02057	0.03409	0.04942	0.02043	0.00705	0.00061
N×P	*M. chinensis*	0.07341	0.02566	0.05549	0.03800	0.03910	0.00539	0.00050
N×P	*T. lanceolata*	0.08227	0.02184	0.05780	0.02619	0.02383	0.00902	0.00116
N×P	*E. nutans*	0.04090	0.03165	0.03929	0.04435	0.05455	0.02419	0.00495
N×P	*P. saundersiana*	0.08965	0.02523	0.04068	0.05332	0.04034	0.00179	0.00027
N×P	*M. ruthenica*	0.05140	0.03074	0.01494	0.08143	0.04653	0.01921	0.00221
N×P	*S. nigrescens*	0.03268	0.02140	0.06315	0.02805	0.01904	0.00394	0.00183
N×P	*G. straminea*	0.04505	0.01913	0.03885	0.03591	0.02079	0.00687	0.00101
N×P	*A. diplostephioides*	0.03713	0.02585	0.05828	0.04373	0.03365	0.00347	0.00087
N×P	*G. boreale*	0.05688	0.02342	0.05494	0.03685	0.01909	0.01433	0.00139
N×P	*S. pulchra*	0.03420	0.01499	0.02151	0.01828	0.03077	0.00220	0.00560
N×P	*O. kansuensis*	0.04896	0.02444	0.03881	0.03987	0.04366	0.00705	0.00151
